# Recent Advances in the Treatment of Alzheimer’s Disease Using Nanoparticle-Based Drug Delivery Systems

**DOI:** 10.3390/pharmaceutics14040835

**Published:** 2022-04-11

**Authors:** Prashant Poudel, Soyeun Park

**Affiliations:** College of Pharmacy, Keimyung University, Daegu 42601, Korea; prashant.kmu10@gmail.com

**Keywords:** Alzheimer’s disease, nanoparticles, blood–brain barrier, liposomes, dendrimers, polymeric nanoparticles, solid–lipid nanoparticles

## Abstract

Alzheimer’s disease (AD) is an irreversible and progressive neurodegenerative disorder. Most existing treatments only provide symptomatic solutions. Here, we introduce currently available commercial drugs and new therapeutics, including repositioned drugs, to treat AD. Despite tremendous efforts, treatments targeting the hallmarks of AD show limited efficacy. Challenges in treating AD are partly caused by difficulties in penetrating the blood–brain barrier (BBB). Recently, nanoparticle (NP)-based systems have shown promising potential as precision medicines that can effectively penetrate the BBB and enhance the targeting ability of numerous drugs. Here, we describe how NPs enter the brain by crossing, avoiding, or disrupting the BBB. In addition, we provide an overview of the action of NPs in the microenvironment of the brain for the treatment of AD. Diverse systems, including liposomes, micelles, polymeric NPs, solid-lipid NPs, and inorganic NPs, have been investigated for NP drug loading to relieve AD symptoms, target AD hallmarks, and target moieties to diagnose AD. We also highlight NP-based immunotherapy, which has recently gained special attention as a potential treatment option to disrupt AD progression. Overall, this review focuses on recently investigated NP systems that represent innovative strategies to understand AD pathogenesis and suggests treatment and diagnostic modalities to cure AD.

## 1. Introduction

Alzheimer’s disease (AD) is a slowly progressive neurodegenerative disease that has created a huge and expanding health care burden. Over 50 million individuals worldwide are facing disruptive cognitive functions, and the number of newly diagnosed cases is rapidly increasing due to increases in the human life expectancy and the aging of populations [1,2]. AD is the sixth-leading cause of death in the United States, as official death certificates recorded 121,499 deaths from AD in 2019. Based on updated calculations, the number of people older than 65 years with Alzheimer’s dementia is projected to reach 12.7 million by 2050 [3]. The total cost to care for and treat AD patients in 2020 was estimated to be $305 billion, which is expected to increase to more than $1 trillion as the population ages [4]. 

Research topics related to AD span a vast spectrum. Many new drugs are under clinical investigation for cognitive enhancement, neuropsychiatric mitigation, and symptom-modifying treatment of AD. These developments are both propitious and disappointing at the same time. Currently, the most common strategy for treating AD is to prescribe cholinesterase inhibitors and N-methyl-D-aspartate (NMDA) receptor antagonists. According to the US Food and Drug Administration (FDA), four different medicines have been approved for the treatment of AD: donepezil in 1997, rivastigmine in 2000, galantamine in 2001, and memantine in 2003. However, these medicines do not correlate with the fundamental pathology of AD [5]. Aducanumab, a monoclonal antibody approved by the US FDA in June 2021, is the only disease-modifying therapeutic (DMT) for the treatment of AD. This drug is designed to act on the deposition of amyloid-β (Aβ) plaques and neurofibrillary tangles in the brain [6]. Despite many attempts to understand and treat AD, there are many hurdles to overcome due to the ambiguity in efficient pathological targets and the necessity for early treatment of AD [7]

First, AD therapeutics must enter the brain by overcoming the blood–brain barrier (BBB). BBB is one of the most specialized biological barriers in the human body. The pathogenesis of AD includes BBB dysfunction, which leads to the failure of Aβ transport from the brain to the peripheral circulatory system, causing neuroinflammation and oxidative stress [8]. Moreover, the BBB is the first protective defense line to prevent foreign substances from traversing from the blood to the brain. Three principal pathways—“crossing,” “avoiding,” and “disrupting” the BBB—can be exploited to overcome the BBB and promote drug delivery to the brain. First, drugs can cross the BBB by exploiting multiple pathways such as transcellular and paracellular pathways. Second, avoiding the BBB describes all other routes of administration that escape direct physical interaction with the BBB and exploit local delivery routes such as intracerebroventricular, intrathecal, intracerebral, and surgical routes. Third, disruption of the BBB exemplifies non-invasive methods such as focused ultrasound sonication, radiation, and the use of hyperosmotic agents and surfactants. The evolution of nanoparticle (NP)-based approaches such as liposomes, micelles, quantum dots, chitosan, dendrimers, and gold NPs may be a promising solution for brain delivery [9]. 

Various NPs have been investigated as targeted and controlled drug delivery vehicles. The key technological assets of NPs are their high stability, high drug loading capacity, diversity in incorporating both hydrophilic and hydrophobic compounds, and versatility in administration routes, including oral application and inhalation [10]. Some studies have reported that hydrophilic NPs with particle sizes less than 100 nm are capable of avoiding opsonization [11], thereby achieving prolonged circulation and the ability to accumulate on specific target sites [12].

Optimal pathological targets can be selected by adopting a particular nano-drug delivery approach. For instance, Topal et al., targeted ApoE to deliver solid lipid NPs with donepezil via cholinergic pathways [13]. Aβ is depleted by selenium-rich nanoparticles (Se-NPs), which reduce the production of reactive oxygen species (ROS) [14]. In addition, biodegradable polymeric NPs with appropriate surface modifications have successfully delivered various diagnostic and therapeutic substances for AD [15]. Polymeric NPs are able to open tight junctions in the BBB and provide loaded substances with a camouflaging membrane barrier, which protects drugs from enzymatic degradation and achieves sustained drug release [16]. 

In this review, we highlight novel NP-based approaches to advance the development of precision AD therapeutics. We also provide an overview of recent pipeline drugs to increase the awareness and understanding of new innovative strategies for the treatment and management of AD patients.

## 2. AD Pathology

AD is a disease whose detailed pathogenesis remains unclear. There are two fundamental hallmarks of AD pathology. First, the aggregation of fragments, that is, Aβ plaques, are found at the outer membrane of neurons. In addition, there are twisted strands of the tau protein, called tau tangles, in the cytosol of neurons. Several hypotheses regarding AD pathology have been reported, as explained below [17]. 

### 2.1. Cholinergic Deficit

During the progression of AD, cholinergic neurons in the basal forebrain are critically damaged. Histo-pathologically, there are decreases in the number of neurons and the marker enzymes responsible for acetylcholine (ACh) synthesis and degradation. Neurochemically, this cerebral cholinergic deficit leads to memory loss and other cognitive and non-cognitive symptoms, which are characteristics of AD [18,19]. Cholinergic dysfunction is a well-known characteristic of AD; however, cholinesterase inhibitors such as donepezil, galantamine, and rivastigmine only relieve the symptoms but not the progression of AD. These clinical observations demonstrate that cholinergic imbalance may only be a clinical feature of AD. Fundamental treatment requires a better understanding of the etiology of AD [20,21].

### 2.2. Amyloid Hypothesis

The abnormal processing of amyloid precursor protein (APP) by β- and γ-secretases produces extracellular non-vascular accumulation of Aβ40 and Aβ42 peptides [22,23]. These peptides have a high binding affinity that makes them chemically sticky and bond with the neuronal membrane, which leads to plaque formation. The clumps of Aβ monomers disrupt the signaling between neurons, causing impaired brain function, which initiates the immune response leading to inflammation and causing damage to surrounding neurons [24]. Aβ aggregation is also responsible for triggering the conversion of normal tau into a toxic state. [25,26,27].

### 2.3. Tau Hypothesis

Tau is a highly soluble microtubule-associated protein (MAP) that prevents the dissociation of tracks in microtubules [28]. Hyperphosphorylation of tau caused by various physiological and metabolic abnormalities such as peculiar Aβ, tau kinase, brain glucose, and the expression of mutated isoforms results in the formation of neurofibrillary tangles (NFTs) [29,30,31]. Insoluble NFTs hinder cytoplasmic functions, deteriorates transportation across axons, and thus leads to neuronal cell death [32]. It is also evident that toxic tau associated with NFTs increases Aβ toxicity via a feedback loop [26].

### 2.4. Oxidative Stress

Reactive oxygen species (ROS) such as hydrogen peroxide radicals (H_2_O_2_), hydroxide radicals (OH^-^), and superoxide radicals (O_2_^-^) are the byproducts of the mitochondrial electron transport chain (ETC) and are the main contributors to the oxidative damage and death of neurons [33]. In particular, the brain uses more oxygen than other tissues and undergoes mitochondrial respiration, which increases the likelihood of exposure to ROS. AD is highly associated with cellular oxidative stress, including increased protein oxidation, protein nitration, lipid peroxidation, glycol oxidation, and Aβ accumulation, and Aβ can likewise induce oxidative stress [34,35,36].

### 2.5. Cellular and Vascular Dysfunction 

Advancing age and vascular risk factors are two biological events that contribute to a high risk for both AD and vascular dementia [37,38]. Cerebral microvascular pathology and cerebral hypoperfusion can stimulate degenerative and cognitive changes in patients with AD. Different types of cerebrovascular deficiency, such as reduced blood flow to the brain or impaired microvascular integrity in cortical regions, can occupy preliminary or intermediate positions in a series of events that result in cognitive impairment causing AD [39]. The critically attained threshold of cerebral hypoperfusion (CATCH) causes dysregulation in the production of endothelial nitric oxide (NO), leading to capillary degeneration [40].

### 2.6. Cholesterol

Cholesterol regulates the generation and clearance of Aβ. A variant of the apolipoprotein E (APOE) gene is consistent with the role of cholesterol in the pathogenesis of AD and has been identified as a major genetic risk factor for AD [41,42]. Many *in vitro* and *in vivo* studies on AD have demonstrated that elevated levels of cholesterol expedite Aβ formation [43,44]. Intracellular cholesterol is also involved in the regulation of APP processing by directly modulating secretase activity [45].

### 2.7. Inflammation

AD neuroinflammation involves inflammatory components, pathways of complement activation, and other receptors in brain cells, such as microglia and astrocytes [46]. Aβ can stimulate microglia and astrocytes to express major histocompatibility complex II (MHC II) and increase the secretion of interleukins, prostaglandins, leukotrienes, thromboxanes, coagulation factors, and protease inhibitors. These responses orchestrate the inflammatory response in patients with AD [47,48]. In contrast, the B cell receptor CD22 has a negative effect on phagocytosis and causes microglial dysfunction. CD-22 suppression facilitates the removal of Aβ fibrils from the brain, which could be a viable strategy to reduce the progression of neuroinflammation and AD [49].

### 2.8. Metal Imbalance

Metal ion imbalance induces Aβ and tau pathologies by directly or indirectly affecting intracellular pathways, whereas homeostatic disruption conversely worsens metal ion transport/deposition [50]. It promotes Aβ overproduction through activation of β- or γ-secretase and inhibition of α-secretase [51]. It also causes hyperphosphorylation of tau through the activation of protein kinases, including glycogen synthase kinase-3β (GSK-3β), mitogen-activated protein kinases (MAPKs), cyclin-dependent protein kinase 5 (CDK5), and inhibition of protein phosphatase 2A (PP2A) [52]. Amyloid plaques consist of high levels of metal components, such as copper (Cu), iron (Fe), and zinc (Zn) [53]. Cu and Fe cooperate with Aβ to induce oxidative stress [54,55,56]. Recent studies have also provided evidence that Zn^2+^ is responsible for Aβ deposition [57,58]. Therefore, metal chelation is a potential way to ameliorate AD pathologies and provides a new direction for AD treatment [59].

## 3. Commercially Available Medicines and Those in Pipelines

Tacrine was the first drug approved by the FDA in 1993 for the treatment of mild to moderate AD. Liu et al., demonstrated that tacrine disrupts the trafficking of acetylcholinesterase in the endoplasmic reticulum, resulting in neuronal apoptosis [60]. However, it was withdrawn from the market in 2013 owing to concerns related to its hepatotoxicity. Currently, commercially available therapeutics for the treatment of AD have been developed to inhibit several pathologic targets, including cholinesterase inhibitors (e.g., donepezil, galantamine, and rivastigmine), N-methyl-D-aspartate (NMDA) receptor antagonists (e.g., memantine), and a monoclonal antibody that directly disrupts Aβ (aducanumab), as shown in Table 1. Aducanumab was launched in the market through an accelerated approval program in the US FDA in 2021 as an AD therapeutic. In addition, there are many ongoing clinical trials to develop new therapeutics to treat AD. Promising candidates in the pipeline for the treatment of AD are summarized in Table 2.

## 4. BBB Physiology and Structure

The BBB is a formidable doorkeeper that protects the brain from foreign substances, including pathogens. However, it is a major obstacle in the management of neurological disorders, including AD and Parkinson’s disease (PD), because it prohibits many potential therapeutics from penetrating into the brain [61,62]. BBB dysfunction is directly correlated with amyloid and tau pathology in AD [8]. Recently, drug-loaded NPs have been actively investigated as precision medicines that enter the brain by binding to target proteins associated with the BBB [63]. Because AD is a neurodegenerative disease of the CNS, drugs used to treat AD must pass through the BBB, otherwise they will fail to achieve the expected efficacy, regardless of their action mechanism. Thus, permeability through the BBB is an indispensable prerequisite for therapeutic agents to treat AD. 

**Figure 1:**
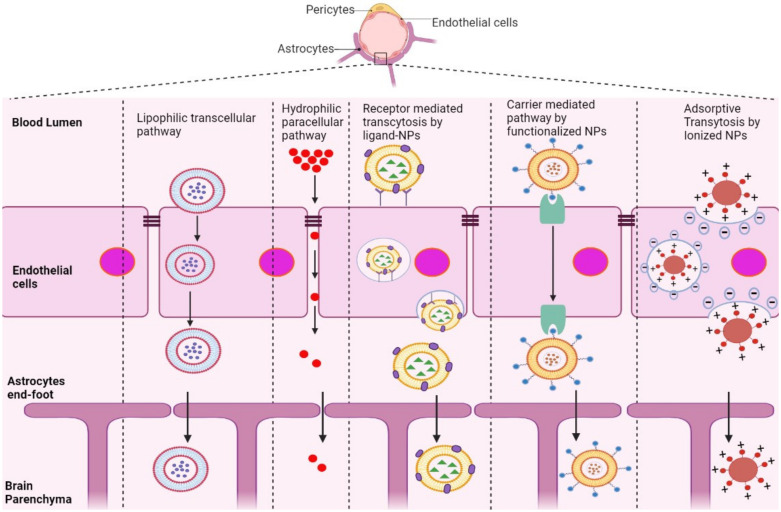
Schematic representation of potential pathways of NP-based drug delivery systems that penetrate the BBB for the treatment of AD.

The BBB is composed of multiple cell types, including endothelial cells (ECs), astrocytes, microglial cells, and pericytes. Transportation across the BBB can be achieved via both transcellular and paracellular pathways (Figure 1). Transcellular pathways play a pivotal role in transporting lipophilic agents, while paracellular pathways transport hydrophilic molecules [64]. Transcellular pathways involve endothelial carrier-mediated transport (CMT), receptor-mediated transcytosis (RMT), adsorptive-mediated transcytosis (AMT), and active efflux. CMT is responsible for the transportation of vitamins, hormones, organic anions/cations, and nucleotides across the BBB. CMT involves spontaneous passive transport via transmembrane integral proteins along a concentration gradient. The brain always needs glucose, so transport will occur even if blood glucose levels are low via glucose transporters such as GLUT-1 and GLUT-3 (Table 3). Different amino acids can also cross the BBB through the CMT pathway; large neutral amino acids such as levodopa, histidine, tryptophan, and valine are transported via LAT1 and excitatory amino acids, such as glutamate and aspartate, are transported via excitatory amino-acid transporters EAATs (Table 3). Large molecules such as antibodies, transferrin, insulin, and low-density lipoprotein (LDL) are transported through RMT. RMT is the primary pathway for the transport of macromolecules that are critical for brain function across the BBB. It involves transporters such as insulin receptor (IR), insulin-like growth factor receptor (IGFR), type 1 transferrin receptor (TfR1), low-density lipoprotein receptor (LDLR), leptin receptor (LEPR), and neonatal Fc receptor (FcRN) (Table 3). AMT involves electrostatic interactions between cationic and anionic molecules on the endothelial cell membrane, which aids in the transport of cationic proteins and basic oligopeptides, such as cell-penetrating peptides. Under these circumstances, efflux pumps such as glycoprotein-P (PgP), ATP-binding cassette transporters (ABC), and multidrug resistance proteins create a resistant barrier that prevents drugs from passing through the BBB. However, aging or pathological conditions can increase the influx of certain drugs into the brain. Paracellular pathways allow the transmission of constituents by penetrating the intercellular space across the epithelium. However, transcellular transport allows constituents to travel through a cell by crossing both the apical and basolateral membranes. There are three major types of transporters responsible for BBB penetration, which can be utilized as targets to deliver therapeutics into the brain, as shown in Table 3. 

## 5. Strategies to Overcome the BBB

### 5.1. Crossing the BBB

NPs used to deliver drugs across the BBB are as small as 1–100 nm in size. NPs are adapted to overcome several hurdles faced by drugs in treating AD [65]. Many therapeutics for AD distribute throughout the body, with few reaching the brain, where it is primarily required due to the tightly organized BBB. Recently, various types of NPs have been shown to enhance the targeting efficiency to the brain and reduce the toxicity and adverse effects caused by the systemic circulation of a drug throughout the body [66]. 

RMT is the process by which NPs enter the brain microenvironment by taking advantage of overexpressed receptors in the BBB. This pathway induces endocytosis via clathrin-coated pits or caveolae, similar to AMT [67]. After NPs are internalized, they can comply with distinct cellular pathways, relying on their size, composition, charge, and ligand conjugation [68]. Most NPs with conjugated ligands, such as lactoferrin, transferrin, ApoE, and B6-peptide, are mediated by receptors, including LR, TfR, LDLR, IR, and IGFR, as shown in Table 3.

Positively charged NPs in contact with the negatively charged plasma membrane of the BBB undergo AMT [69,70]. This mechanism is stimulated by electrostatic interactions between the cationic groups of ligands conjugated on the surface of NPs and negative moieties present on the luminal surface of endothelial cells [70]. Positively charged ligands, such as lectin, cardiolipin, heparin, and CPPs, are functionalized on the surface of NPs, inducing BBB adsorption and transcytosis of the nanocarrier into the brain [71].

NPs conjugated with molecules including glucose, mannose, glutathione, and amino acids, which are recognized by transporters overexpressed in endothelial cells of the BBB, are mediated by CMT [72]. This involves transporters such as GLUT1, GLUT3, LAT1, and EAAT (Table 3), which covalently bind to glucose and mannose and ease penetration of nanocarriers into the BBB [73,74,75]. However, the small and stereospecific pores involved in CMT interfere with the uptake of large molecular drugs [76].

### 5.2. Avoiding the BBB 

AD therapeutics can reach the CNS via local delivery routes without physically interacting with the BBB. Specifically, this pathway includes administrative routes that avoid passage through the BBB, such as injection either through surgical exposure to the brain by means of a catheter or pump (intracerebroventricular, intraparenchymal/intracerebral administration) or through spinal puncture (intrathecal administration). Intracerebral administration is a direct method of drug delivery, although drug diffusion is only possible if the target sites are adjacent to the parenchymal surface or ventricles. This route has been shown to be inefficient and clinically restricted [77,78]. However, Zhu et al., recently formulated angiopep-2-modified lipid-coated mesoporous silica NPs loaded with paclitaxel (ANG-LP-MSN-PTX) to target glioma. When blood–brain microdialysis was conducted to study their pharmacokinetic behavior, the half-life and AUC_blood_ of ANG-LP-MSN-PTX displayed sustained systemic delivery via the intracerebral route of administration [79]. Likewise, Yurek et al., prepared DNA NPs that were injected intracerebrally into the denervated striatum of a rat model and showed significantly higher transgene expression in the brain [80]. 

Intracerebroventricular (ICV) and intrathecal procedures are performed by direct injection into the skull and ventricles or puncturing the lumbar region. Generally, the ICV route is used for injecting chemical compounds and peptides, such as colchicine, okadaic acid, streptozotocin, scopolamine, Aβ_1-42_, and hyperphosphorylated tau (p-tau), directly into the cerebral lateral ventricles to induce AD in different experimental animal models [81,82,83]. The intrathecal route accesses the subarachnoid space (SAS), where the cerebrospinal and interstitial fluids of the parenchyma are exchanged, which results in delivery to the entire CNS. Householder et al., intrathecally administered PEG-NPs into healthy mice and showed rapid distribution through the SAS with a reproducible pattern of brain delivery [84]. Polyamidoamine (PAMAM) dendrimers administered through the intrathecal route were also observed to be localized in activated microglia and astrocytes responsible for neuroinflammation. This method ensures the targeted delivery of therapeutics to the sites of diseases such as AD, cerebral palsy, and multiple sclerosis [85]. Despite the fact that they can deliver an enormous number of drugs to the CNS, these procedures are very intrusive and convey a significant risk of CNS infection or neurotoxicity [86]. For these reasons, intranasal (IN) routes that bypass the BBB via the sensory organs are preferred.

IN drug delivery is a non-invasive brain-targeting alternative because the brain and nose compartments are associated through olfactory, trigeminal, and systemic circulation. Among these, systemic circulation is only partially involved, reducing the risk of systemic side effects. Advantages of the IN route include ease of administration, high patient compliance, and rapid delivery kinetics within minutes [87,88]. The actual mechanism of IN drug delivery is not yet clear, but there is robust evidence that supports the significance of olfactory and trigeminal nerve pathways because of their direct and immediate connection from the nose to the brain [89]. For example, astaxanthin-loaded solid lipid NPs (SLNs) demonstrated a compelling neuroprotective effect against oxidative stress in PC-12 cell lines. IN delivery of the 99mTc-labeled SLN in experimental rats showed enhanced brain uptake, as measured by gamma scintigraphy and radiometry [90]. Curcumin is a natural anti-inflammatory and antioxidant phytochemical compound with the potential to treat AD, however, it has poor water solubility and limited BBB penetration. Mesoporous silica NPs (MSNP) were adapted to evaluate their efficacy in the nose-to-brain delivery of curcumin. Desirable loading efficiency, uptake, and release of this phytochemical were reported for curcumin-loaded MSNPs in a neuroblastoma cell line [91]. Similarly, tarenflurbil failed in phase III trials due to its poor BBB penetration; however, when incorporated into poly(lactic-co-glycolic acid (PLGA) NPs and SLNs and delivered intranasally, its overall pharmacokinetics were improved and the nose-to-brain route was observed to be superior to IV and oral administration [92]. 

### 5.3. Disrupting the BBB

Another strategy for overcoming the BBB involves opening the BBB through force using non-invasive physical methods, such as focused ultrasound sonication (FUS), and the administration of biochemical reagents, including cell-penetrating peptides (CPP), hyperosmotic agents, and surfactants. 

FUS combines systematically circulating microbubbles embedded with NPs to locally improve vascular permeability and treat AD [93]. Microbubbles (MBs) are small drug delivery vehicles filled with gas that are typically between 0.5 μm and 10 μm in diameter, which is generally too bulky to enter the BBB. FUS is used to disrupt tight junctions and create openings in a specific localized area, resulting in enhanced BBB permeability [94]. Liu et al., prepared quercetin-modified sulfur NPs embedded in microbubbles (Qc-SuNPs-MB), which were demolished immediately upon exposure to ultrasonic waves. The sonoporation effect allowed the opening of the BBB and the release of Qc-SuNPs from the outer shell of the MB, which accumulated in the brain microenvironment. The rapid accumulation of Qc-SuNPs in the brain parenchyma effectively reduced the inflammatory response, neuronal death, oxidative stress, and calcium homeostasis imbalance, thereby treating AD [95]. Kafoed et al., investigated the transgene distribution and immune response of recombinant adeno-associated virus (rAAV) incorporated into MBs. Intravenous MBs of rAAV, when combined with FUS, triggered an immune reaction, including MHC class II expression, T cell infiltration, and microglial activation [96]. 

CPP facilitates the rapid internalization of exogenous cargo, such as proteins, nucleic acids, liposomes, and other NPs. Cheng et al., designed a co-delivery strategy in which positively charged CPP (TAT) and negatively charged plasmids were linked by electrostatic interactions and then incubated with positively charged mesoporous silica NP (MSN) to deliver curcumin, promote neuronal growth, and reduce oxidative stress [97]. 

Chemical compounds such as bradykinin induce vasodilation, resulting in enhanced BBB permeability [98]. Visible to near-infrared light (NIR) irradiation has likewise been exploited in several studies to further develop NP-based brain delivery, as NIR applications increase the BBB opening through the local heating effect and prompt a temporal disruption of the BBB [99]. A nanogel system made up of carbonyl-functionalized poly(N-vinyl pyrrolidone) produced by ionizing radiation was covalently bonded to insulin, serving as a nanocarrier that was efficiently transported across the BBB for the treatment of AD [100]. Zhou et al., designed a nanocomposite using hollow nano-ruthenium (Ru) as a carrier, which was loaded with nerve growth factor (NGF) and sealed with a phase change material (PCM). Under NIR radiation, the nanocomposite produced an excellent photothermal effect as it effectively penetrated the BBB and released NGF, which inhibited tau hyperphosphorylation and aggregation, reduced oxidative stress, and restored nerve damage, ultimately improving memory in an AD-induced mouse model [101]. Likewise, NIR application was used to propel the motion of the Janus nanomotor (JNM-I), which demonstrated an increased BBB permeability and promoted contact between the Aβ fibrils and the Aβ inhibitors [102]. Although these strategies provide new alternatives to overcome the BBB, it is not certain whether translational, repetitive, and prolonged BBB opening might also induce neurotoxicity [86,103]. 

## 6. NP-Based Delivery System for the Treatment of AD

Table 4 provides an overview of various recent developments in NPs that have been adapted in the field of AD therapeutics. We highlight various types of NPs, drug molecules they are loaded with, components of carrier materials, conjugated ligands on the surface of NPs, drug doses, route of administration, type of model, and outcomes observed in different *in vitro*, *in vivo*, and *ex vivo* research models. Particle size and zeta potential, which greatly affect their stability in suspension, adsorption into the BBB, and endocytotic uptake, are summarized in Table 4 [104,105,106]. 

**Figure 2:**
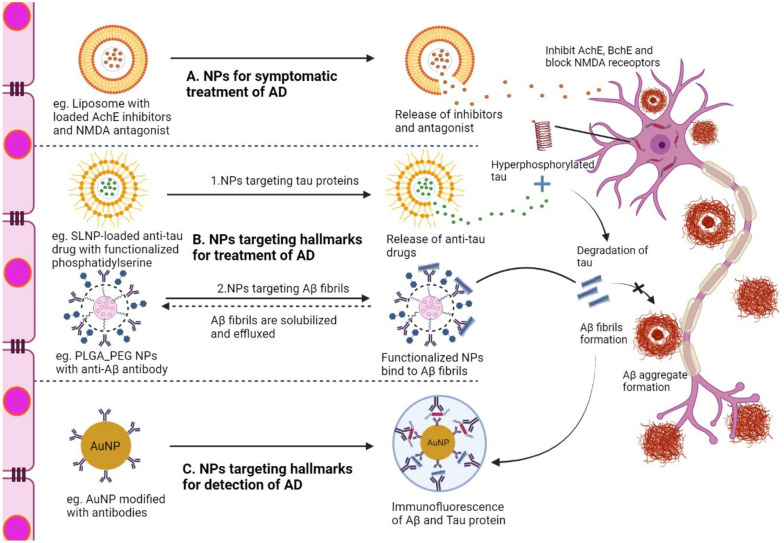
Illustration of the action mechanism of NPs in neurons associated with AD after overcoming the BBB. (**A**) Liposome with loaded AchE inhibitors targeting cholinergic system impairment, (**B**) 1. SLNP-loaded anti-tau drug with functionalized phosphatidylserine targeting hyper-phosphorylated tau proteins, (**B**) 2. PLGA-PEG with anti-Aβ antibody is involved in targeting, solubilizing, and clearing Aβ fibrils, (**C**) Modified AuNP with capture antibody targeting Aβ and tau proteins, making a sandwich with secondary antibody for diagnosing hallmarks associated with AD. AchE, acetylcholinesterase; NMDA, N-methyl D-aspartate antagonist; Aβ, amyloid beta fragment, PEG, polyethylene glycol; SLNP, solid lipid nanoparticle, NP, nanoparticle; PLGA, poly D,L-lactic-co-glycolic acid; AuNP, gold nanoparticle.

Figure 2 represents the potential mechanism of action of various NPs in diseased neurons associated with AD. The mechanisms of NPs are mainly classified into three categories: NPs for symptomatic treatment, NPs targeting the hallmarks of AD, and NPs targeting hallmarks for the diagnosis of AD. First, NPs loaded with AchE inhibitors and NMDA antagonists (donepezil, rivastigmine, galantamine, memantine) (Table 4) reach the target site and release the cargo, which inhibits enzymes that enhance cholinergic transmission and blocks the current flow through channels of NMDA, relieving the symptoms of AD. Second, NPs loaded with drugs targeting the hallmarks of AD (Aβ, tau) act as disease-modifying therapeutics that have the potential to cure AD. The anti-tau drugs (LY3372689, TRx0237) (Table 2) target hyperphosphorylated tau proteins and inhibit tau protein aggregation. In addition, NP-conjugated anti-Aβ antibody (aducanumab, pipenimab) (Table 2) captures Aβ fibrils and effluence in the blood circulation, thereby reducing Aβ plaques. Third, modified NPs with conjugated capture antibodies and functionalized fluorescence can provide images of Aβ fibrils and tau proteins, which can have a major impact on the diagnosis of AD.

### 6.1. Liposomes

Liposomes are spherical, double-layered microscopic vesicles that consist of phospholipid bilayer membranes. The inner compartment of the liposomes can be used to deliver hydrophilic payloads. Commercially available AD therapeutics such as galantamine, rivastigmine, and donepezil have shown efficient penetration and enhanced bioavailability when loaded into liposomes [107,108,109] (Table 4) (Figure 2). In contrast, the outer lipid layers are suitable for delivering lipophilic drugs. In addition, the outer layer acts as a shielding barrier until the liposomes reach the target site. They are sometimes conjugated with targeting moieties to precisely and proficiently enter the brain through the BBB [110]. Arora et al., demonstrated efficient brain-targeted delivery of ApoE2-encoding plasmid DNA (pApoE2) using surface-modified liposomes for effective AD therapy [111]. Saffari et al., also formulated phosphatidylserine-based liposomes loaded with metformin, which showed higher efficacy in improving learning and memory deficits and decreasing neuroinflammation in AD-induced rat models than free metformin [112] (Table 4). Glutathione-targeted PEGylated liposomes enhanced the brain delivery of amyloid-targeting antibody fragments across the BBB into the brain [113]. Kong et al., reported that the use of PEGylated liposomes induced osthole to cross the BBB and accumulate in the brain, thus alleviating the AD-related pathology in APP/PS-1 mice [114]. Pardridge incorporated plasmid DNA as a gene therapy for Trojan horse liposomes (THLs) to enhance the delivery of DNA to the brain [115]. THLs are prepared by incorporating thousands of strands of PEG polymer, where the tips of 1–2% of the PEG strands are conjugated with peptide-or receptor-specific monoclonal antibodies that act as molecular trojan horses, triggering receptor-mediated transcytosis of the THL across the BBB [116]. Liposomes have also been used to detect amyloid protein aggregation in AD. Kocsis et al., mounted multiple dye units on the surface of a vesicle, which increased the binding affinity for α-synuclein fibrils to enhance the detection of amyloid aggregates [117]. 

### 6.2. Micelles

Micelles are spherical amphiphilic drug carriers with a particle size in the 5–50 nm range, which have a hydrophilic shell and a hydrophobic core, each of which plays a specific role. The hydrophilic shell makes micelles water-soluble and enables intravenous delivery, while the hydrophobic core carries the treatment drugs. Yang et al., prepared neuronal mitochondria-targeted micelles (CT-NM) with loaded resveratrol, resulting in stabilization of the dynamic balance between mitochondrial fission and fusion, which restored cognitive performance in APP/PS1 transgenic AD mice [118]. Increased neuroinflammation of the BBB has been observed due to the expression of the receptor for advanced glycation end-products (RAGE), causing AD [119]. A polymeric micelle drug delivery system (AB-PEG-LysB/CUR) was constructed with three components, including a RAGE-targeting peptide (AB) originating from Aβ protein, an amphiphilic polymer (PEG-LysB) with ROS responsiveness and scavenging ability, and the hydrophilic drug curcumin targeting Aβ aggregation (Table 4). This micelle accumulated in the diseased regions through the Aβ transportation-mimicked pathway and exhibited synergistic effects of polymer-based ROS scavenging and cargo-based Aβ inhibition upon microenvironmental stimulation [120]. In addition, in a study performed on an aluminum chloride-induced AD animal model, lactoferrin-conjugated linoleic acid (LF-CLA) micelles displayed increased biodistribution in the brain and cognitive capabilities and decreased brain oxidative stress, inflammation, apoptosis, and acetylcholine esterase activity [121]. Geng Hao et al., prepared conjugated polymer-based thermoresponsive micelles (CPMs), which revealed a robust capability to capture lethal Aβ aggregates at biological temperatures [122].

### 6.3. Solid Lipid NPs (SLNs) 

SLNs are solid-state lipid-based network systems 50–1000 nm in diameter with a lipid-forming core at both room and physiological temperatures. These SLNs permit high entrapment of hydrophobic drugs with a controlled release profile. At present, SLN has emerged as a reputed drug carrier system that efficiently transports active pharmaceutical cargo across the BBB to specific target sites in the brain. This novel approach is beneficial because of the controlled drug delivery, longer circulation time, target specificity, higher efficacy, and more importantly, reduced toxicity. Misra et al., formulated galantamine hydrobromide-loaded SLNs, which offered enhanced *in vitro* drug release (>90% during 24 h) and also displayed a substantial memory restoration capacity in cognitive-deficit AD rodents [123]. The size and entrapment efficiency of galantamine in SLNs were ~100 nm and 83%, which was similar to galantamine loaded in liposomes. However, the formulated NPs were administered orally as SLNs and intranasally as liposomes. *In vivo* pharmacokinetic studies showed that oral SLNs had ~30 times greater AUC than intranasal liposomes loaded with galantamine [107]. Shah et al., formulated SLNs by homogenization and ultrasonication using Compritol 888 ATO, Tween-80, and poloxamer-188 as the lipid, surfactant, and stabilizer, respectively, for effective intranasal delivery of hydrophilic rivastigmine [124]. Additionally, Chauhan and Sharma invented transdermal patches with rivastigmine-loaded nanolipid carriers for the treatment of dementia [125]. The encapsulation efficiencies of lipid NP prepared by Shah and Chauhan were 66.84 ± 2.49% and 70.56 ± 1.20%, respectively, which is about two times higher than the rivastigmine-loaded liposomes prepared by Yang et al., which was 30.5 ± 8% [108].

*In vitro* studies of donepezil-loaded lipid gels demonstrated effective skin permeation and enhanced transdermal delivery of donepezil for the treatment of AD [126]. Different release patterns of donepezil were observed when comparing transdermal SLNs and intranasal liposomes loaded with donepezil. The initial burst release of the intranasally administered liposomal formulation was observed at 150 min, whereas transdermally administered SLN were observed at 12 h. This indicates that the donepezil-loaded SLN exhibited sustained release, whereas the donepezil-loaded liposomes exhibited immediate release. Recently, SLNs loaded with the hematopoietic factor erythropoietin (SLN-EPO) were used to improve memory deficits in an AD-induced rat model. Erythropoietin (EPO) is a promising neuroprotective candidate with a high molecular weight and hydrophilicity, that is rapidly cleared from circulation, and thus, hardly penetrates the BBB. However, EPO-loaded SLNs enhance BBB penetration, prevent Aβ plaque deposition and antioxidant properties, and reduce the ADP/ATP ratio [127]. Vakilinezhad et al., prepared phosphatidyl (PS)-functionalized SLNs loaded with nicotinamide to treat AD-induced rats (Table 4). The nicotinamide-loaded SLNs exhibited better memory improvement, conserved more neuronal cells, and minimized tau hyperphosphorylation in experimental rats compared to a non-formulated conventional administration in the early stage of AD [128]. Resveratrol, a polyphenolic compound that exerts neuroprotective effects, displays poor solubility, a short biological half-life, and rapid metabolism and elimination, leading to low bioavailability. However, when incorporated with SLNs, its stability is extended for a longer period, and its bioavailability is enhanced. As a result, resveratrol incorporated with SLNs showed enhanced efficacy by ameliorating oxidative stress and cognitive deficits in vascular dementia by activating the Nrf2/HO-1 pathway [129,130]. Transferrin-functionalized SLNs of quercetin were formulated to enhance the targeting efficiency as well as antioxidant activity, which eventually relieved AD symptoms [131,132]. *In vitro* and *in vivo* studies of lactoferrin-modified SLNs loaded with curcumin provided evidence of increased targetability and activity in the brain for the treatment of AD [133].

### 6.4. Polymeric NPs (PNPs)

PNPs are solid vehicles produced from natural or synthetic polymetric materials with nanosized colloidal organic compounds. Recently, a wide spectrum of polymers has been investigated for the purpose of designing PNPs capable of targeted delivery of drugs for the treatment of AD [134]. PLGA is the most widely investigated polymer because of its controlled and sustained-release properties, low toxicity, and biocompatibility with cells and tissues. PLGA has been approved by both the US FDA and the European Medicine Agency (EMA) for use in vaccines, drug delivery, and tissue engineering [135]. Baysal et al., showed that donepezil-loaded poly(lactic-co-glycolic acid)-block-poly (ethylene glycol) [PLGA-b-PEG] NPs destabilize Aβ fibril formation *in vitro* and yield a neuroprotective effect. Unlike liposomes and SLNs loaded with donepezil, it has been shown that 60% of the drug loaded in PLGA-b-PEG was released in the first 2 h and its release reached a plateau, exhibiting a controlled release profile [109,126,136]. In addition, hybrid PNPs with poly(L-lactic acid) (PLA) and PLGA-based delivery systems have produced extended-release formulations that can reduce dosing frequency and minimize side effects. For instance, Nanaki formulated PLA/PLGA hybrid NPs with loaded galantamine, which showed successful intranasal delivery in AD-induced rodent models [137]. The size of the galantamine-loaded PNP was ~200 nm, which is twice as large as that of galantamine-loaded liposomes (~100 nm), while the zeta potential of the galantamine-loaded liposomal NP was −49 mV compared to PNP (−17 mV), which shows that they are less prone to aggregation [107]. Pager et al., prepared L-lactide-depsipeptide PNPs using a single emulsion-solvent evaporation method. These PNPs were loaded with rivastigmine and achieved sustained release for 72 h, which led to a higher efficacy with improved safety [138]. Resveratrol incorporated into PNPs (RES-PNPs) prepared by amphiphilic methoxy-polyethyleneglycol-poly-caprolactone (mPEG-PCL) block copolymers showed a significant improvement in the antioxidative ability of resveratrol. Animal studies using RES-PNPs showed evidence of alleviating injury from γ-ray radiation and toxicity of Aβ peptide overexpression in *Caenorhabditis elegans* through DPPH radical scavenging and over-regulation of SOD-3 expression [139]. Fan et al., incorporated curcumin, a compound that has anti-amyloid, anti-inflammatory, and antioxidant properties for the treatment of AD in PLGA-PEG PNPs conjugated with B6 peptide. These PNPs were non-cytotoxic in HT22 cells and were able to cross the BBB *in vitro* and *in vivo*. Histological studies using these PNPs demonstrated that they reduce hippocampal Aβ formation as well as tau hyperphosphorylation [140]. Similarly, PEG-PLA NPs modified with B6 peptide were also prepared using an emulsion solvent evaporation technique. The resulting B6-PNPs displayed a desirable biodistribution profile with significantly increased cellular accumulation. Several studies have demonstrated that B6-modified NPs are capable of accumulating in brain capillary endothelial cells via clathrin-mediated and lipid raft-mediated endocytosis. The encapsulation of neuroprotective peptide-NAPVSIPQ (NAP) in B6-NPs showed outstanding amelioration of learning impairments, cholinergic disruption, and loss of hippocampal neurons in AD-induced transgenic mice [141]. Cano et al., suggested a dual drug therapy using epigallocatechin-3-gallate (EGCG) and ascorbic acid (AA) loaded in PLGA-PEG PNPs. *In vitro* and *ex vivo* experiments of EGCG/AA PNPs demonstrated that they disrupted tight junctions and opened up the BBB. Moreover, *in vivo* studies in AD-induced mice reduced Aβ plaques and neuroinflammation, and enhanced synaptogenesis, memory, and learning processes [142]. Pioglitazone (PGZ), an approved drug for type-2 diabetes, has emerged as a promising candidate for AD treatment because it shows neuroprotective properties and acts as a peroxisome proliferator-activated receptor (PPARγ) agonist. The solvent displacement technique was adopted to synthesize PGZ-PNPs using PLGA-PEG as a carrier to cross the BBB. Moreover, they demonstrated a reduction in Aβ deposition as well as memory deficits in AD-induced transgenic mice [143]. Sun et al., prepared and characterized quercetin-encapsulated biodegradable PLGA NPs using a double emulsion solvent evaporation technique. These quercetin PLGA NPs inhibited the cytotoxicity caused by zinc (Zn^2+^) Aβ, improved cognition and memory impairments, increased the therapeutic index, and decreased side effects [144]. 

Chitosan is also a biodegradable and biocompatible polymer that is regarded as a promising PNP-based carrier for brain drug delivery. Chitosan NPs have some unique features, such as a mucoadhesive nature and intrinsic bioactivity, which can not only promote penetration of drugs into the brain through the olfactory route, but also represent anti-Alzheimer therapeutics themselves [145]. Intranasal administration of thiolated chitosan PNPs loaded with galantamine contributed to the significant recovery of amnesia-induced mice by improving *in vivo* memory-augmenting effects and inhibiting acetylcholinesterase activity. The mucoadhesive property of formulated thiolated chitosan PNPs increased their retention at the olfactory mucosa, resulting in an increase in the permeability of galantamine by opening tight protein channels [146]. Some studies have also demonstrated that chitosan is an effective chelating agent capable of competing with Aβ or histidine for copper binding [147]. This competition demonstrates the fundamental chemical interactions involving copper-Aβ-chitosan, and highlights the association between the copper complex and neurodegenerative diseases, suggesting possibilities for a new target for the treatment of these diseases.

### 6.5. Dendrimers

Dendrimers are regularly branched molecules with well-defined and multivalent 3D morphology. The solitary functional unit of a dendrimer that germinates branches is called a dendron. The cargo drugs are either attached to the surface of the dendrimer or enclosed in their branches. Gothwal et al., showed that the administration of dendrimers formed by polyamidoamine (PAMAM) and lactoferrin (Lf) conjugates loaded with memantine (MEM-PAMAM-Lf) in aluminum chloride-induced AD rats improved cognition, behavioral patterns, and memory [148] (Table 4). A recent report showed that a novel dendrimer-PPARα/γ dual agonist conjugate (D-tesaglitazar) induced an ‘M1 to M2′ phenotype shift and increased Aβ phagocytosis of macrophages. [149]. Interestingly, the positively charged dendrimers consisting of PAMAM, poly(propylene imine) (PPI), and poly-l-lysine showed dose-dependent toxicity. However, negatively charged dendrimers, such as sulfonated, carboxylated, phosphonated, or neutral dendrimers, are less toxic than positively charged dendrimers. Klajnert et al., demonstrated that a gallic acid-triethylene glycol (GATG) dendrimer conjugated with 27 terminal morpholine groups ([G3]-Mor) significantly reduced Aβ fibril formation [150]. 

### 6.6. Nanoemulsions (NEs)

NEs are kinetically stable nanosized isometric mixtures of oil and aqueous phases stabilized by surfactant or co-surfactant molecules to form a single phase. They are prepared by high-pressure homogenization, ultrasonication, and an emulsion inversion point [151]. NEs are utilized to improve drug delivery to the targeted sites and alleviate adverse effects and toxic reactions. The type of oil used in NEs plays a pivotal role in their uptake into the CNS. In addition, the lipidic nature of NEs imparts a high potential to deliver the cargo drug to the brain through the BBB [152,153]. Recently, Kaur et al., prepared NEs with loaded memantine and donepezil for the treatment of AD. Both memantine and donepezil-loaded NEs showed dose-dependent toxicity on the Neuro 2a cell line. *In vivo* studies of both memantine and donepezil-loaded NEs administered via intravenous, intranasal, and oral routes in AD-induced rats demonstrated that intranasal administration resulted in maximum uptake in the brain compared to other administration routes [154,155]. The NE loaded with memantine showed a faster drug release rate of 80% in 6 h, similar to the rate of 77% at 6 h observed for the memantine-loaded dendrimers. However, dendrimers loaded with memantine exhibited a positive zeta potential (20 mV), while NE exhibited a negative potential (−19.6 mV) [148,154]. However, the NE-loaded donepezil exhibited higher zeta potential (−10.7 mV) in comparison to liposome, SLN, and PNP [109,126,136]. In contrast, huperazine (Hup A), a selective acetylcholinesterase inhibitor, was loaded with lactoferrin (Lf)-conjugated NEs (Table 4). Jiang et al., showed that Hup A-loaded NEs exhibited a significant increase in BBB penetration through intranasal administration, and thus, enhanced the action duration, targeting ability, and safety [156].

### 6.7. Inorganic NPs

Inorganic nanoparticles include a broad range of substances, including gold, silver, aluminum, and silicon dioxide. They are non-poisonous, hydrophilic, biocompatible, and exceptionally stable compared to organic materials. They have gained substantial consideration in preclinical development as potential diagnostic and therapeutic systems for a variety of applications, including imaging, drug delivery, and the development of radiotherapy [157]. These NPs mainly include quantum dots (QDs), gold NPs (Au NPs), and magnetic NPs (MNPs). 

QDs are nanocrystals of semiconducting particles that are 1.5–10 nm in diameter. These NPs acquire unique properties, including size-dependent optical properties, superior photostability, a large Stokes shift, and high extinction coefficient and brightness. Recently, Tak et al., synthesized graphene QDs from the flower of *Clitoria ternatea* (ctGQDs), which showed greater inhibition of acetylcholinesterase compared to donepezil [158] and were effective in reducing the AD-like symptoms in rodents (Table 4). Another study also found that nontoxic and biocompatible black phosphorous QDs (BPQDs) significantly inhibited insulin aggregation and deteriorated Aβ sheets by inhibiting amyloid aggregation [159]. Recently, Zhou et al., reported large amino acids that imitate selenium-doped carbon QDs (SeCQDs) as novel nano-based agents for the multi-targeted treatment of AD. The functionalized α-carbonyl and amino groups on the edge of SeCQDs significantly lessen Aβ aggregation and restrain neurodegeneration in AD rodents [160]. Mars et al., also prepared curcumin-graphene QDs (Cu-GQDs) to sense ApoE4 DNA, which is responsible for AD. The dual electrochemical and fluorescence-sensing of Cu-GQDs exhibited high selectivity, stability, and sensitivity with a 4.7% relative standard deviation (RSD) toward targeted ApoE4 DNA, even in human blood plasma [161].

AuNPs are colloidal gold particles that are 1–100 nm in diameter. They are used in thermal ablation, highly sensitive diagnostic assays, radiotherapy enhancement, and drug delivery. Recently, Hou et al., designed AuNPs loaded with stabilized L- and D-glutathione, which crossed the BBB by intravenous administration and inhibited Aβ42 without noticeable toxicity [162]. Tramontin et al., evaluated the efficacy of intracerebroventricularly injected AuNPs with 100 µg of okadaic acid (OA) in an AD animal model. Interestingly, AuNPs without any loaded drug prevented tau phosphorylation, restored oxidant levels in the brain, and prevented neuroinflammation [163]. In a similar study, the neuroprotective role of bare AuNPs was demonstrated through intrahippocampal and intraperitoneal routes in an AD-induced rat model. The AuNP led to substantial improvements in the acquisition and retention of spatial learning and memory, along with enhanced neuronal survival indicated by the articulation of various brain-derived neurotrophic factors (BNDF), cAMP response element-binding protein, and stromal interaction molecules such as STIM1 and STIM2 [164]. The AuNP-based immunosensor is also used to detect tau, a hallmark of AD [165]. Another important hallmark of AD, Aβ oligomers, can also detected using an electrochemiluminescent aptasensor with the enhancement of signals by AuNPs conjugated with a metal–organic framework (AuNP/MOF) [166].

MNPs consist of two parts: a magnetic material, which is frequently nickel, iron, and cobalt, and a functional chemical component. Recently, MNPs have attracted great attention due to their appealing properties in the use of nanomaterial-based catalysts, biomedicine, and magnetic resonance imaging. Superparamagnetic iron oxide nanoparticles (SPIONs) have been modified as dual diagnostic and therapeutic (theranostic) systems for several neurodegenerative diseases, including AD. SPIONS yielded a 10-fold higher bioavailability of quercetin in the brain of an AD animal model than free quercetin [167]. Recently, Li et al., successfully formulated antibiofouling polymer polyethylene glycol-block-allyl glycidyl ether (PEG-b-AGE) magnetic iron oxide nanoparticles (IONPs) for the detection of Aβ peptides and tau protein in AD. The prepared IONPs efficiently suppressed non-specific interactions with Aβ peptides and tau proteins, and when conjugated with the capturing antibody, showed improved specificity (>90%) and sensitivity (>95%). Thus, antibody-conjugated magnetic nanoparticles for liquid biopsy of AD can exhibit better performance in capturing Aβ peptides and tau proteins [168]. MNPs are promising theranostic carriers for the treatment of AD because of their excellent biocompatibility and simple functionalization. MNPs were encapsulated with memantine labeled with fluorescein (Mem-Flu) in sodium dodecyl sulfate micelles to form a magnetic nanoemulsion (MNE). The prepared MNE gained the capacity for fluorescence imaging of Aβ1-42 peptide plaques-fibrils [169]. Yin et al., synthesized sialic acid (SA)-modified selenium (Se) NPs conjugated with B6 peptide (B6-SA-SeNPs), which were capable of preventing Aβ-induced cell toxicity in PC12 and bEnd.3 cells. Moreover, B6-SA-SeNPs effectively penetrate the BBB and accumulate in the targeted cells [170]. Multifunctional magnetite NPs loaded with small interfering RNA (siRNA) were synthesized for the potential treatment of AD. The co-immobilization of translocational outer membrane protein A (OmpA) on PEGylated magnetite NPs enabled cellular internalization and significantly boosted the endosomal escape property of the prepared vehicle for siRNA without affecting its biocompatibility. These results suggest that OmpA/PEGylated NPs exhibit enhanced cellular uptake via the endocytic pathway and successfully suppress the BACE1 gene in HFF-1 cells without cytotoxic effects [171].

## 7. Immunotherapy for AD

The immune system plays a significant role in AD pathogenesis. AD is an autoimmune disease characterized by progressive memory deficits, cognitive impairment, and a paradigm shift in personality [172]. Amyloid senile plaques, neurofibrillary tangles, and neuroinflammation are histopathological hallmarks of progressive synaptic dysfunction and, ultimately, neuronal apoptosis [173]. Impaired microglia are also considered risk factors for AD. There is increasing evidence that microglia prevent the occurrence of AD, as a disorder in microglial activity and changes in microglial response to Aβ are associated with increased AD risk. On the contrary, there is considerable evidence that activated microglia can cause neuronal damage. Normally, microglia act as housekeeping phagocytes that maintain tissue homeostasis and maintain the extracellular space free of Aβ, thereby protecting against AD. As the level of Aβ increases, microglia engulf and clear Aβ aggregates, and when Aβ activity is removed, microglia condense Aβ into dense plaques and restrict them from neurons. These latter preventive activities are supported by ApoE, which relies on TREM2 and activates microglia into the disease-associated microglia (DAM) state [174,175]. Fassbender et al., successfully illustrated the connection between innate immune receptors and AD. As fibrillar Aβ is responsible for inducing cellular activation, they found that CD14, a lipopolysaccharide (LPS) receptor, interacts only with fibrillar but not with nonfibrillar Aβ. This interaction intercedes Aβ-induced microglial and monocytic activation and neuronal toxicity. Since AD brains become incorporated with a high concentration of Aβ fibrils for many years, this interaction is sufficient to maintain chronic neuroinflammation [176]. Several immunotherapies are already in clinical trials and several more are likely to be initiated in the future. Aβ immunotherapy, including the active and passive administration of anti-Aβ antibodies, reduced Aβ accumulation and prevented AD-like pathology in a transgenic mouse model. Active immunization with intact Aβ_42_ peptide stimulates the immune response of T-cells, B-cells, and microglia, whereas active immunization with synthetic Aβ fragments conjugated with carrier protein stimulates T-cells to offer cytokines that permit B-cells to mature and produce antibodies. Passive immunization with monoclonal anti-Aβ antibodies directed against Aβ avoids the need for AD patients to initiate an immunological response to the Aβ peptide [173]. As Aβ immunotherapy develops into meningoencephalitis, researchers are investigating other possible interventions to treat AD. Tau lesions are more strongly associated with dementia than Aβ plaques; therefore, their removal may be clinically more effective than the removal of Aβ [177]. Anti-tau antibodies (tau Ab) may be phagocytosed by microglia in an isotype-dependent manner. However, the optimal immunoglobulin (Ig)-G isotype for therapeutic tau Ab has yet to be explored in detail, which might be vital for tau clearance [178]. Immune checkpoint inhibitors also enhance the systemic adaptive immune response that might combat neurodegeneration in AD [179,180]. For instance, Baruch et al., demonstrated that an immune checkpoint blockade directed against the PD-1 pathway induced interferon (IFN)-γ-dependent activity. The observed systemic immune response prompted the recruitment of monocyte-derived macrophages leading to the removal of cerebral Aβ plaques and enhanced cognitive performance in the five familial AD mutations (5XFAD) mouse model [179]. Likewise, Rosenzweig et al., also found that blocking PD-1 demonstrates similar efficacy in both 5XFAD mice and in mice with the human tau gene with two mutations associated with front temporal dementia (DM-htau). The results showed that a PD-1 immune checkpoint blockade is advantageous in both Aβ and tau models of AD, activating a universal immune repair mechanism [181]. However, many investigations have failed to produce a convincing effect of immune checkpoint blockades in AD-induced mouse models [182,183]. Intriguing evidence suggests that the receptor for advanced glycation end products (RAGE) binds to soluble Aβ circulating across the BBB, implicating the development of AD progression [184]. The RAGE/Aβ interaction stimulates microglial activation and produces oxidative stress, which causes neuronal damage [185]. Thus, RAGE inhibitors are currently being tested as potential treatments for AD patients [186]. 

Nanotechnology-based drug delivery systems are promising tools for immunotherapies to improve nuclear entry, overcome obstacles in delivery, prevent *in vivo* degradation and enhance uptake in target cells. For instance, immunoliposomes are widely used to selectively target cancer cells tumor-specific antigens. Loureiro et al., designed PEGylated liposomes functionalized with two monoclonal antibodies (mAbs), anti-transferrin receptor mAb, and anti-Aβ mAb, for AD. *In vivo* studies in wild-type rats demonstrated that dual-ligand conjugation promotes efficient drug delivery to the brain [187]. CLVFFA, a central hydrophobic fragment of Aβ, when conjugated as a hexapeptide at the surface of gold nanoclusters (AuNCs), acts as a nano-sized inhibitor (AuNCs-CLVFFA). Thioflavin T fluorescence and transmission electron microscopy demonstrated that AuNCs-CLVFFA inhibited Aβ_40_ fibrillogenesis, fibril prolongation, and fibrils disaggregation [188]. Similarly, NPs can be used as immunotherapeutic cargos for the treatment and diagnosis of AD. Skaat et al., formulated NIR-fluorescent iron oxide NPs conjugated with anti-Aβ mAb that inhibited Aβ_40_ fibrillation kinetics and selectively detected these fibrils *ex vivo* by both MRI and fluorescence imaging [189]. In addition, dopamine (DA)-functionalized CuInS_2_/ZnS quantum dots (QDs) were used as fluorophores in a novel tyrosinase-conjugated anti-tau Ab sandwich fluorescence immunoassay. The DA-functionalized CuInS_2_/ZnS QDs rapidly detected tau proteins, revealing a new method to detect disease biomarkers [190]. 

## 8. Future Perspectives and Conclusions

Sustained increases in the prevalence of AD surely grab the attention of disease control authorities. AD is the most common neurodegenerative disease, and it contributes to the mortality rates in aged populations. It is increasingly evident that new therapeutics and treatment modalities are required, considering the progressive challenges posed by neurological diseases. Although numerous investigations have proposed new disease-modifying drugs, a lack of precise remedies for the treatment of neurodegenerative diseases remains. Most studies that have demonstrated successful outcomes in preclinical studies have failed to reproduce acceptable results in clinical trials. Most treatment strategies focus on justifying AD symptoms as opposed to targeting pathophysiological factors. However, a limited number of target-acting moieties, gene therapies, and nerve regeneration therapies target the causative factors for removal. In addition, Aβ and tau immunotherapies for AD unite two complex biological mechanisms: the immune system and the nervous system. Progress in immunotherapy in preclinical models of AD has shown great potential to exceed both treatment and diagnostic approaches, revealing further possibilities for the management and prevention of AD. The recent FDA approval of aducanumab (an anti-Aβ monoclonal antibody) for AD treatment has provided hope that new drugs that can fundamentally cure AD and other neurodegenerative diseases will be produced. 

One of the reasons for the slow progress in the development of AD therapeutics is the BBB. Although many strategies to overcome the BBB and reach the brain have shown considerable efficiency, the brain delivery efficiency (e.g., dose percentage/AD brain) in some AD-induced animal models has not been quantitatively established and needs to be further investigated. In addition, cellular neurovascular unit models using endothelial cells derived from patients may provide a better understanding of various BBB strategies. Recently, the study of increased integrin and adhesion molecules and leukocyte diapedesis have become interesting targets to be investigated. However, these studies are still in their infancy and thus require further research [191,192]. 

For these reasons, NPs are a promising modality because of their enhanced ability to penetrate the BBB and their theranostic function in the treatment of AD. Different types of NPs such as liposomes, SLNs, PLGA-PLA NPs, AuNPs, and QD have been designed to improve therapies for AD. Significant surface functionalization can be achieved with these NPs to improve BBB penetration, drug-loading capacity, targetability, and bioavailability. These NPs can encapsulate AD therapeutics, such as donepezil, rivastigmine, galantamine, memantine, and conjugate antibodies on the surface of NPs. However, many issues still need to be addressed, such as the accurate determination of the transition time, location, degree, and diversity of different NPs crossing the BBB. Real-time quantitative monitoring of different NPs will also be informative for choosing appropriate NPs to penetrate the BBB for drug delivery and to optimize the therapeutic regimen for AD patients. At the same time, it is equally important to evaluate whether the biological activity of the drug loaded in NPs is retained or improved. NPs easily aggregate and form clusters that may block blood vessels. In recent studies, metal-containing NPs such as AuNPs, QDs, and iron oxide NPs, despite their advantages, have been shown to induce the activation of glial cells, generation of ROS, release of inflammatory factors, and apoptosis, causing neurotoxicity. These NPs can induce BBB damage or dysfunction of memory, cognition, and learning [193]. As it is difficult to penetrate the BBB with NPs, it is not easy for certain NPs to be eliminated by clearance systems and can accumulate in the brain and cause cytotoxicity [194]. 

Thus, future studies using NP-based delivery to the brain should focus on improving safety, precise targetability, and pharmaco-kinetic features. The safety and effectiveness of particular NPs should be evaluated in human clinical trials to identify the most encouraging and cost-effective AD therapeutics. Comprehensive handling and administration protocols must be developed for the safe utilization of NPs by patients and healthcare professionals. In addition, the theranostic development of NPs is crucial for the potential treatment of AD, which brings significant advances in precision and personalized medicine. These personalized therapies focus on assessing the efficacy and safety of the treatment progress, which significantly improves the clinical outcome of an individual AD patient. 

Finally, it can be concluded that NP-based delivery systems have a significant therapeutic potential for AD. Considering the advantages of crossing the BBB and treatment approaches, including symptomatic relief, DMT therapy, and hallmark diagnosis, further translational research to explore the interplay of NPs in a biological interface should be performed. The combined application of NPs and newly discovered drugs will undoubtedly provide promising options for the treatment and diagnosis of AD in the future. 

## Figures and Tables

**Table 1:** Commercially available drugs for the treatment of AD.

Drugs	Trade Name (Company)	Action Mechanism	Dosage Form	Dose	LogP (Permeability) Molecular Weight (M.W) (g/mol)	K (Partition Coefficient)
Tacrine	Cognex (Sciele, Atlanta, GA, USA)	Reversible inhibition of acetylcholinesterase (AChE)	Capsule	Initial dose: 10 mg orally q.i.d (between meals if possible) for 6 weeks. Maintenance dose: may increase to 20 mg orally q.i.d. Further increases to 120 mg and 160 mg/day may be done in 6-week intervals.	2.71 M.W = 234.72	
Donepezil	Aricept (Pfizer, New York City, NY, USA)	Reversible inhibitor of acetylcholinesterase which prevents the hydrolysis of acetylcholine.	Tablet	5 mg q.d., may increase to 10 mg/day after 4–6 weeks if tolerated, then to 23 mg/day after at least 3 months	4.7 M.W = 379.5	log K_ow_ = 4.86 (est)
Rivastigmine	Exelon (Novartis, Basel, Switzerland)	Inhibits the hydrolytic activity of AChE and BChE and binds to catalytic sites.	Solution Capsule	Oral Solution and capsules 6 mg to 12 mg per day, administered b.i.d. (daily doses of 3 mg to 6 mg b.i.d.)	2.3 M.W = 250.34	
Galantamine	Razadyne (Janssen, Beerse, Belgium)	Binds reversibly to acetylcholine esterase and enhances the intrinsic action of acetylcholine on nicotinic receptors.	Tablet Capsule Oral solution	Tablets contain 4 mg, 8 mg, and 12 mg galantamine. Capsules contain 8 mg, 16 mg, and 24 mg galantamine. Oral solution contains 4 mg galantamine (as 5.13 mg galantamine hydrobromide) per mL.	1.8 M.W = 287.35	
Memantine	Namenda (Allergan, Dublin, Ireland)	N-methyl D-aspartate (NMDA) antagonist	Tablet Oral solution	Tablets: 5 mg q.d., may increase to 10 mg/day, 15 mg/day, and 20 mg/day at 1-week intervals if tolerated Oral solution: same as above	3.28 M.W = 179.3	log K_ow_ = 3.28
Memantine and Donepezil	Namzaric (Actavis, Parsippany, NJ, USA)	N-methyl D-aspartate (NMDA) antagonist plus cholinesterase inhibitor	Capsule (ER)	7 mg memantine/10 mg donepezil q.d., may increase to 28 mg memantine/10 mg donepezil in 7 mg increments at 1-week intervals if tolerated	M.W = 215.76	
Aducanumab	Aduhelm (Biogen, Cambridge, MA, USA)	Targets accumulated Aβ plaques	Intravenous infusion	Doses 1 and 2 (weeks 0 and 4)—1 mg/kg IV over one hour Doses 3 and 4 (weeks 8 and 16—3 mg/kg IV over one hour	M.W = 145,912.34	

**Table 2:** Promising candidates in pipeline to treat AD.

	Drug	Mechanism of Action	Therapeutic Purpose	Stage of AD	Target	Phase	Sponsor	Start Estimated End Dates
	Disease-Modifying Biologics							
New Therapeutics	Aducanumab	Disrupts Aβ plaques and oligomers	DMT	Mild to moderate	Amyloid	3	Biogen, Cambridge, MA, USA	Mar 2020 Oct 2023
	Gantenerumab	Monoclonal antibody acts at Aβ plaques and oligomers	DMT	Mild to moderate	Amyloid	2	Roche, Basel, Switzerland	Dec 2020 Feb 2024
	Pepinemab	Monoclonal antibody directed at semaphoring 4D to reduce inflammation	DMT	Mild	Inflammation	1	Vaccinex. Inc., Rochester, NY, USA	Jul 2021 Jan 2023
	LY3372689	Tau protein aggregation inhibitor	DMT	Moderate	Tau	2	Eli Lilly & Company, Indianapolis, IN, USA	Sep 2021 Jun 2024
	TRx0237	Inhibition of tau protein aggregation	DMT	Mild to moderate	Tau	3	TauRx Therapeutics, Aberdeen, Scotland	Jan 2018 Jun 2023
	Disease-Modifying Small Molecules							
	Sumifilam	Alters conformation of filamin A	DMT	Mild to moderate	Filamin A	2	Cassava Sciences, Inc, Austin, TX, USA	Nov 2021 Oct 2023
	Tricaprilin (contains caprylic acid triglyceride)	Induces ketosis and improves mitochondrial and neuronal function	DMT	Mild to moderate	Metabolism and bioenergetics	3	Cerecin, Anson Road, Singapore	Jan 2021 Feb 2023
	Symptoms-Reducing Small Molecules							
	AD-35	Acetylcholinesterase inhibitor	Cognitive enhancer	Mild to moderate	Neurotransmitter receptors	2	Zhejiang Hisun Pharmaceutical, Jiaojiang District, Taizhou, China	Dec 2018 July 2021
	Disease-Modifying Biologics							
Repositioned Drugs	BCG vaccine	Vaccination against tuberculosis infection; immunomodulator	DMT	-	Inflammation/immunity	2	Mindful Diagnostics and Therapeutics, Eau Claire, WI, USA	Nov 2020 Dec 2021
	IVIG (NewGam 10%)	Polyclonal antibody	DMT	Mild	Amyloid	2	Sutter Health, Sacramento, CA, USA	Jan 2011 Dec 2019
	Disease-Modifying Small Molecules							
	Losartan & amlodipine & atorvastatin + exercise	Angiotensin II receptor blocker (losartan), calcium channel blocker (amlodipine), cholesterol agent (atorvastatin)	DMT	-	Vasculature	3	University of Texas Southwestern, Houston, TX, USA	Feb 2017 Mar 2022
	Metformin	Sensitize insulin to improve metabolism of CNS glucose	DMT	Mild	Metabolism and bioenergetics	3	Columbia University, NIA, NY, USA	Jan 2021 Apr 2024
	Montelukast	Leukotriene receptor antagonist	DMT	Mild to moderate	Inflammation	2	Emory University, Atlanta, GA, USA	Sep 2019 Oct 2022
	Hydralazine	Antioxidant	DMT	Mild to moderate	Oxidative stress	3	Shahid Sadoughi University of Medical Sciences and Health services, Yazd, Iran	Jun 2021 Jun 2023
	Suvorexant	Dual orexin receptor antagonist; improves sleep with effects on CSF Aβ	DMT	-	Neurotransmitter receptors	2	Washington University School of Medicine, St. Louis, MO, USA	Jan 2021 Jan 2025
	Trehalose	Induces autophagy and promotes clearance of aggregated proteins	DMT	Mild to moderate	Cell death	1	Mashhad University of Medical Sciences, Mashhad, Razavi Khorasan, Iran	Aug 2020 Aug 2022
	Vorinostat	Histone deacetylase (HDAC) inhibitor; enhanced synaptic plasticity	DMT	Mild	Epigenetics	1	German Center of Neurodegenrative Disease (DZNE), Bonn, Germany	Sep 2017 Mar 2022
	Symptoms-Reducing Small Molecules							
	Caffeine	Pleiotropic effect on CNS function	Cognitive Enhancer	Mild	Metabolism and bioenergetics	3	University Hospital, Lille, France	Nov 2021 Nov 2024
	Escitalopram	Selective serotonin reuptake inhibitor	Neuropsychiatric symptoms	Mild	Neurotransmitter receptors	3	Johns Hopkins University, NIA, Baltimore, MD, USA	Jan 2018 Aug 2022
	Nabilone	Synthetic cannabinoid; antiemetic	Neuropsychiatric symptoms (agitation)	Mild to moderate	Neurotransmitter receptors	3	Sunnybrook Health Sciences Center, ADDF, Torinto, ON, Canada	Feb 2021 Oct 2025
	Nicotine	Nicotinic acetylcholine receptor agonist	Cognitive enhancer	Mild	Neurotransmitter receptors	2	University of Southern, California, NIA, ATRI, Vanderbilt University, Nashville, TN, USA	Jan 2017 July 2023
	Prazosin	α-1 adrenoreceptor antagonist	Neuropsychiatric symptoms (agitation)	Mild to moderate	Neurotransmitter receptors	2	ADCS, NIA, Maitland, FL, USA	Oct 2018 Dec 2022
	Riluzole	Glutamate modulator agent	Cognitive enhancer	Mild	Neurotransmitter receptors	2	Icahn School of Medicine at Mount Sinai, NY city, NY, USA	Completed
	Sargramostim	Recombinant human GM-CSF	Cognitive enhancer	Mild to moderate	Metabolism and bioenergetics	2	University of Colorado, Denver, USA	Dec 2021 Jul 2024
	THC-free CBD oil	Cannabinoid with effects on cannabinoid receptors	Neuropsychiatric symptoms agents (agitation)	Severe	Neurotransmitter receptors	2	Eastern Virgina Medical School, Norfolk, VA, USA	Feb 2021 Jun 2022

ADCS, Alzheimer’s Disease Cooperative Study; ADDF, Alzheimer’s Drug Discovery Foundation; ATRI, CA, California; DMT, Disease-modifying therapy; GM-CSF, Granulocyte–Macrophage colony-stimulating factor; NIA, National Institute on Aging.

**Table 3:** Various transporters that penetrate the BBB.

Influx Transporters	Efflux Transporters	Receptor Transporters
Choline transporter (ChT)	Peptide transport system-6 (PTs-6)	Insulin receptors (IR)
Sodium-coupled glucose transporters (SGLTs)	Breast cancer resistant protein (BCRP)	Insulin-like growth factor receptor (IGFR)
Cationic amino acid transporter (CAT1)	P-glycoprotein (P-gp)	Transferrin receptors (TfR)
1-type large amino-acid transporter (LAT1) Excitatory amino-acid transporters (EAATs)		Leptin receptor (LepR)
Glucose transporter (GLUT1) (GLUT3)		Low-density lipoprotein receptor (LDLR)
Monocarboxylate lactate transporter (MCT1) Receptor for glycation end products (RAGE)		Neonatal Fc receptor (FcRN)
		Lactoferrin receptor (LR)

**Table 4:** Nanoparticle-based drug delivery systems to treat AD.

Drug	Carrier Material	Ligand	Particle Size (nm)	Zeta Potential (mV)	Route of Administration, Dose	*In Vitro/In Vivo* Model	Outcome	Reference
Liposome								
Metformin	Phosphatidyl serine	-	145	−41	Intraperitoneal, 50 mg/kg	Adult male Wistar rats	*in vivo:* Decreased neuroinflammation. Enhanced cognition restoration.	[112]
-	Chitosan	pApoE2	167.8 ± 2.47	19.8 ± 3.6	Intravenous, 1 mg/kg	bEnd.3 cells C57BL/6 mice	*in vitro*: Decreased cell viability in all cell lines. Increased concentration of phospholipid. *in vivo*: Approximately 2 times higher ApoE protein expression than endogenous ApoE levels.	[111]
Osthole	-	Transferrin	104.28 ± 3.76	−6.95 ± 0.56	Tail vein intravenous, 10 mg/kg	hCMEC/D3 cells and APP-SH-SY5Y cells APP/PS-transgenic mice	*in vitro/in vivo*: pH-controlled release up to 72 h. Enhanced penetration and drug accumulation.	[114]
	GSH-PEG	VHH-pa2H Glutathione (GSH)	108		IV bolus injection, 5 mg/kg	APP_swe_/PS1dE9 transgenic mice	*in vivo*: Increased standard uptake values (SUV) of VHH-pa2H in the blood.	[113]
Galantamine HBr	Soya Phosphatidylcholine	-	112 ± 8	−49.2 ± 0.7	Oral and intranasal, 3 mg/kg	PC-12 cell, male SD rats	*in vitro*: Reduced cytotoxicity Enhanced BBB penetration. *in vivo*: More bioavailability in brain. Enhanced efficacy.	[107]
Donepezil	1,2-distearyl-sn-glycero-3-phospholine (DSPC)	-	102 ± 3.3	−28.31 ± 0.85	Oral and intranasal, 1 mg/kg	Male Wistar rats	*in vitro*: 75.5% drug release in 8 h. *in vivo*: Enhanced penetration. Enhanced bioavailability.	[109]
Rivastigmine	EPC, Cholesterol, DSPE-PEG-CPP	CPP	178.9	−8.6 ± 2.4	Intranasal and intravenous, 1 mg/kg	Endothelial cells, male SD rats	*in vitro* High encapsulation efficiency. Sustained-release behavior. *in vivo* IN route shows more activity	[108]
Micelles								
Resveratrol	PEG-PLA	C3 peptide	43.85 ± 0.94	12.9 ± 0.17	Intravenous, 10 mg/kg	HT22 cells, APP/PS1 transgenic mice	*in vitro*: Improvement on brain accumulation and increased targeting. *in vivo*: Enhance cognitive performance. Inhibited Aβ aggregation. Enhanced activity.	[118]
Curcumin	PEG	Aβ peptide	65		Intravenous	SH-SY5Y, APP_swe_/PS1dE9 transgenic mice	*in vitro/in vivo*: Increased efficacy. Inhibited Aβ aggregation. Improved memory behavior.	[120]
-	Linoleic acid	Lactoferrin	120 ± 12.4	−32.8 ± 3.66	Oral, 4 gm/mL	Adult male Wistar rats	*in vitro/in vivo* Enhanced cognition Reduced oxidative stress. Reduced Aβ aggregation.	[121]
	PMO-b-PBM, POEG-b-PBM and PF	-	70		-	PC-12 cells	*in vitro*: Reduced Aβ aggregation. Reduced Aβ fibrillation-induced cytotoxicity.	[122]
Solid–lipid NPs								
galantamine HBr	Glyceryl behnate, pluronic F-127, tween 80	-	88 ± 1.89	−18.75 ± 1.7	Oral route, 2.5 mg/kg	Adult Wistar rats	*in vitro*: Maximum drug entrapment. Drug release > 90% for a period of 24 h. *in vivo*: Enhanced bioavailability 2-fold Memory restoration.	[123]
Rivastigmine	Campritol 888 ATO	-	82.5 ± 4.07	3.20 ± 1.44	-	Franz diffusion cell, goat nasal mucosa	*in vitro*: Higher diffusion with SLN. Ex-vivo: Maximum diffusion was seen with up to 8 h.	[124]
Donepezil	Stearic acid, oleic acid, lecithin, sodium taurodeoxytaurocholate	-	177.05 ± 2.12	−55.35	Transdermal	-	*in vitro*: Increased drug skin permeation. Enhanced drug delivery.	[126]
Rivastigmine	GMS, castor oil	-	134.5 ± 15.1	−11.8 ± 2.24	Transdermal	Albino Wistar rats	*in vitro*/i*n vivo*: Non-irritant. Enhanced bioavailability.	[125]
Erythropoietin	GMS, span 60, span 80, tween 80	-	219.9 ± 15.6	−22.4 ± 0.8	Intraperitoneal, 1250 IU/kg and 2500 IU/kg	Albino male Wistar rats	*in vitro*: 39% of EPO released in first 3 h followed by approximately 90% for 72 h. *in vivo*: Improved memory dysfunction. Enhanced efficacy.	[127]
Nicotinamide	Stearic acid, phospholipon 90G, sodium taurocholate	Phosphatidylserine	124 ± 0.8	−46.1 ± 0.65	Intravenous or intraperitoneal	BCES, SH-SY5Y, adult male Sprague-Dawley rats	*in vitro*: Enhanced BBB penetration. *in vivo*: Neuroprotective potential Improved cognition impairment.	[128]
Resveratrol	Lecithin	-	286 ± 1.47	−17.5 ± 0.23	Oral, 10 mg/kg	Male Sprague-Dawley rats	*in vitro*: 91% sustained release after 24 h. *in vivo*: 4.5 times bioavailability in brain. Effective antioxidant activity.	[129]
Resveratrol and grape extract	Cetyl palmitate, tween 80, tween 20	Anti-transferrin receptor mAb (OX26 mAb)	254 ± 17	−4.0 ± 0.1	-	HBEC	*in vitro*: Efficient cellular uptake. Inhibited Aβ aggregation.	[130]
Quercetin	-	-	152	−20.7	Intravenous, 4.41 mg/kg	Male Wistar rats	*in vitro/in vivo*: 85.73% Drug entrapment efficiency. Effective in crossing BBB. Improvement in memory.	[132]
Lipid NPs								
Quercetin	-	Transferrin	219 ± 13	−28 ± 2	-	hCMEC/D3 cells	*in vitro*: 80–90% of entrapment efficiency Minimum cytotoxicity at BBB cell line.	[131]
Curcumin	PC, cholesterol oleate, glycerol trioleate	Lactoferrin	103.8 ± 0.6	−5.80 ± 0.73	Intravenous, 10 mg/kg	BCECs, SD rats	*in vitro*/*in vivo*: Effective BBB penetration. Long-term leads to enhanced activity.	[133]
Polymeric-NPs								
Galantamine	PLA-PLGA	-	198.00 ± 0.02	−27.42 ± 0.03	Intranasal, 3 mg/kg	Wistar rats	*in vitro*/*in vivo*: Good stability between NP and drug. More bioavailability in the brain.	[137]
Donepezil	PEG-PLGA	-	174 ± 12	−20.45	-	HBMEC and HA cell	*in vitro*: High destabilizing effect on fibril formation. Decreased neuroinflammation.	[136]
Rivastigmine	L-Lactide-depsipeptide	-	142.2 ± 21.3		-	-	*in vitro:* Entrapment efficiency of 60.72 ± 3.72% was obtained. Showed sustained release with 90% of drug for up to 72 h.	[138]
Resveratrol	Methoxy PEG, -caprolactone	-	80.70 ± 7.12	~0 mV	-	*Caenorhabditis elegans,* N2, CF1553, CL4176, and CL1175	*in vitro*/*in vivo:* Enhanced antioxidant properties. Enhanced radical scavenging. Enhanced anti-lipid peroxidation.	[139]
Curcumin	PLGA-PEG	B6 peptide	~100	3.83 ± 0.89	Intraperitoneal, 25 mg/kg	HT22 cells/APP/PS1 transgenic mice	*in vitro*: Enhanced cellular uptake. Possessed good blood compatibility *in vivo*: Improvement in spatial learning and memory. *Ex-vivo*: Reduced Aβ aggregation.	[140]
ECG	PLGA, PEG, ascorbic acid, tween 80	-	124.8 ± 5.2	−15	Oral, 40 mg/kg	BMVECs, APP/PS1, C57BL/6 mice	*in vitro*: Effective in crossing BBB. *in vivo*: Reduced neuroinflammation. Enhance spatial learning and memory.	[142]
Pioglitazone	PLGA-PEG, tween 80	Anti-Aβ antibody	155.0 ± 1.8	−13.0 ± 0.5	Oral, 10 mg/kg	HBEC,hCMEC/D3 cell line, APP/PS1 transgenic mice	*in vitro*: Better BBB penetration. *in vivo*: Reduced Aβ peptide in brain. Improved memory defect.	[143]
Quercetin	PLGA, PVA	-	150		Intravenous, 20 mg/kg	SH-SY5Y cells, APP/PS1 mice, BALB/c nude mice	*in vitro*/*in vivo*: Low cellular toxicity. High cell viability. Inhibited neurotoxicity. Enhanced cognition and memory.	[144]
-	PEG-PLA	B6 peptide	118.3 ± 7.8	−22.65 ± 0.85	Intravenous, 1 mg/kg	bEnd.3 cells, male ICR mice	*in vitro*: Enhanced brain penetration. *in vivo*: Enhanced cognition.	[141]
Galantamine	Thiolated-chitosan NPs	-	149.3	27.2	Intranasal 4 mg/kg	Swiss albino mice	*in vivo*: Effective acetylcholinesterase activity.	[146]
Memantine	PAMAM (dendrimer)	Lactoferrin	131.72 ± 4.73	20.13 ± 0.94	Intraperitoneally, 2 mg/kg	Swiss albino mice	*in vitro*: PAMAM-MEM maximum release concentration was 77.14 ± 6.0% after 6 h. *in vivo*: Improvement in behavioral responses. Enhanced acetylcholinesterase (AChE) activity.	[148]
Nanoemulsions								
Memantine	-	-	~11	−19.6	Intranasal, 5 mg/kg	Neuro 2a, Sprague-Dawley rats	*in vitro/in vivo*: 98% cell viability and sustained its antioxidative potential. Increased bioavailability.	[154]
Donepezil	Labrasol (10%) as oil, CPC (1%) as surfactant in water (80%), glycerol (10%) as co-surfactant		65.36	−10.7	Intranasal, 0.45 mg/kg	Neuro 2a, Sprague-Dawley rats	*in vitro/in vivo*: Maximum drug release of 99.22% in 4 h in PBS. Non-toxic Effective drug delivery.	[155]
Huperazine A	Capryol 90 (oil phase), cremophor EL & labrasol (surfactant & co-surfactant) & lactoferrin (targeting ligand)	Lactoferrin	16.75 ± 0.4	5.67 ± 0.39	Intranasal	hCMEC/D3 cells, adult Wistar rats	*in vitro/in vivo*: No nasal mucosal toxicity. Effective BBB penetration. Enhanced activity.	[156]
Quantum Dots								
-	Graphene QDs	-	10 ± 1.3	−40 ± 0.4	-	Adult male Wistar rats	*in vitro/in vivo*: Enhanced penetration across BBB. Improved learning and memory. Reduced level of lipid peroxide and nitric oxide.	[158]
-	Black phosphorous QDs	-	~3		-	PC12 cells	*in vitro*: Low cell toxicity. Inhibited insulin and Aβ aggregation.	[159]
-	Selenium-doped carbon QD	-	~25		Intravenous	PC12 cells, adult male Wistar rats	*in vitro/in vivo*: High cell viability. Inhibited Aβ aggregation. Improved memory and cognitive function of an AD rat model.	[160]
Curcumin	Graphene QD & indium-tin-oxide Electrode	-	~8		-	-	*in vitro*: High sensitivity on detection of ApoE4 DNA. Enhanced efficacy.	[161]
Gold nanoparticles								
-	-	L and D glutathione	4		Intravenous, 25 mg/kg	SH-SY5Y cells, C57BL/6 mice	*in vitro/in vivo*: Effective BBB penetration. Improved behavioral performance.	[162]
AuNP	-	-	20		Intraperitoneal, 2.5 mg/kg	Male Wistar rats	*in vivo*: Normalize Tau phosphorylation. Prevented oxidative stress and neuroinflammation.	[163]
AuNP	-	Bucladesine	5	−47.7 ± 10.9	Intrahippocampal, intraperitoneal	Male Wistar rats	*in vivo*: Better acquisition and retention of spatial learning and memory. Improved neuron survival.	[164]
-	3D-Au-PAMAM, electro grafted PABA	CAb-GA conjugate			-	-	Detection: Effective detection of tau protein. LOD value of 1.7 pg/mL.	[165]
Magnetic Nanoparticles								
Quercetin	SPIONs	-	50		Oral, 50 and 100 mg/kg	Male Wistar rats	*in vivo*: Increased penetration. Enhanced Bioavailability.	[167]
-	Sialic acid (SA)-modified selenium (Se) NPs	B6 peptide	95	−14.4	-	PC12 cells and bEnd.3 cells	*in vitro*: Effective in crossing BBB. Inhibitory effects on Aβ peptide.	[170]
siRNA	PEGylated magnetite NPs	OmpA	10		-	HFF-1 cells and SH-SY5Y cells	*in vitro*: Reduced cell toxicity. Enhanced activity. Silencing of BACE1 gene in HFF-1 cells.	[171]

Aβ, amyloid beta fragment; BCEC, brain capillary endothelial cells; Cab-GA, capture antibody crosslinking with glutaraldehyde; CPC, cetyl pyridinium chloride; CPP, cell-penetrating peptide; DSPE, 1,2-distearoyl-sn-glycero-3-phosphorylethanolamine; ECG, epigallocatechin-3-gallate, EPO, erythropoietin, EPC, egg phosphatidylcholine; GSH-PEG, glutathione targeted PEGylated liposome; GMS, glycerin monostearate, HA, haemagglutinin, HBEC, human bronchial epithelial cells; LOD, limit of detection; MEM, memantine; OmpA, outer membrane protein A; PAMAM, polyamidoamine; pApoE2, plasmid DNA apolipoprotein E; PEG-PLA, polyethylene glycol polylactic acid; PF, polyfluorene conjugated polymer; PLGA, poly lactic-co-glycolic acid; PMO-b-PBM, PMO-block-polymerizing with butyl methacrylate;, POEG-b-PBM, POEG-block-polymerizing with butyl methacrylate; PVA, polyvinyl alcohol; SD, Sprague-Dawley rats; SPIONs, superparamagnetic iron oxide nanoparticles; siRNA, small interfering RNA; VHH-pa2H, single-domain antibody fragments.

## Data Availability

Not applicable.

## References

[1] Cummings J., Lee G., Zhong K., Fonseca J., Taghva K. (2021). Alzheimer’s disease drug development pipeline: 2021. Alzheimers Dement. Transl. Res. Clin. Interv..

[2] Alzheimer’s Association (2022). 2022 Alzheimer’s Disease Facts and Figures.

[3] Alzheimer’s Association (2021). 2021 Alzheimer’s disease facts and figures. Alzheimers Dement..

[4] Wong W. (2020). Economic burden of Alzheimer disease and managed care considerations. Am. J. Manag. Care.

[5] Yiannopoulou K.G., Papageorgiou S.G. (2013). Current and future treatments for Alzheimer’s disease. Adv. Neurol. Disord..

[6] Sevigny J., Chiao P., Bussière T., Weinreb P.H., Williams L., Maier M., Dunstan R., Salloway S., Chen T., Ling Y. (2016). The antibody aducanumab reduces Aβ plaques in Alzheimer’s disease. Nature.

[7] Mehta D., Jackson R., Paul G., Shi J., Sabbagh M. (2017). Why do trials for Alzheimer’s disease drugs keep failing? A discontinued drug perspective for 2010-2015. Expert. Opin. Investig. Drugs.

[8] Cai Z., Qiao P.F., Wan C.Q., Cai M., Zhou N.K., Li Q. (2018). Role of Blood-Brain Barrier in Alzheimer’s Disease. J. Alzheimers Dis..

[9] Teleanu D.M., Chircov C., Grumezescu A.M., Volceanov A., Teleanu R.I. (2018). Blood-Brain Delivery Methods Using Nanotechnology. Pharmaceutics.

[10] Gelperina S., Kisich K., Iseman M.D., Heifets L. (2005). The potential advantages of nanoparticle drug delivery systems in chemotherapy of tuberculosis. Am. J. Respir. Crit. Care Med..

[11] Allémann E., Leroux J.C., Gurny R., Doelker E. (1993). *In vitro* extended-release properties of drug-loaded poly(DL-lactic acid) nanoparticles produced by a salting-out procedure. Pharm. Res..

[12] Banerjee T., Mitra S., Kumar Singh A., Kumar Sharma R., Maitra A. (2002). Preparation, characterization and biodistribution of ultrafine chitosan nanoparticles. Int. J. Pharm..

[13] Topal G.R., Meszaros M., Porkolab G., Szecsko A., Polgar T.F., Siklos L., Deli M.A., Veszelka S., Bozkir A. (2020). ApoE-Targeting Increases the Transfer of Solid Lipid Nanoparticles with Donepezil Cargo across a Culture Model of the Blood-Brain Barrier. Pharmaceutics.

[14] Nazıroğlu M., Muhamad S., Pecze L. (2017). Nanoparticles as potential clinical therapeutic agents in Alzheimer’s disease: Focus on selenium nanoparticles. Expert. Rev. Clin. Pharm..

[15] Wong K.H., Riaz M.K., Xie Y., Zhang X., Liu Q., Chen H., Bian Z., Chen X., Lu A., Yang Z. (2019). Review of Current Strategies for Delivering Alzheimer’s Disease Drugs across the Blood-Brain Barrier. Int. J. Mol. Sci..

[16] Sahni J.K., Doggui S., Ali J., Baboota S., Dao L., Ramassamy C. (2011). Neurotherapeutic applications of nanoparticles in Alzheimer’s disease. J. Control. Release.

[17] Hroudová J., Singh N., Fisar Z., Ghosh K. (2016). Progress in drug development for Alzheimer’s disease: An overview in relation to mitochondrial energy metabolism. Eur. J. Med. Chem..

[18] Frölich L. (2002). The cholinergic pathology in Alzheimer’s disease--discrepancies between clinical experience and pathophysiological findings. J. Neural. Transm..

[19] Terry A.V., Buccafusco J.J. (2003). The cholinergic hypothesis of age and Alzheimer’s disease-related cognitive deficits: Recent challenges and their implications for novel drug development. J. Pharm. Exp..

[20] Hampel H., Mesulam M.M., Cuello A.C., Farlow M.R., Giacobini E., Grossberg G.T., Khachaturian A.S., Vergallo A., Cavedo E., Snyder P.J. (2018). The cholinergic system in the pathophysiology and treatment of Alzheimer’s disease. Brain.

[21] Whitehouse P.J. (1998). The cholinergic deficit in Alzheimer’s disease. J. Clin. Psychiatry.

[22] DeTure M.A., Dickson D.W. (2019). The neuropathological diagnosis of Alzheimer’s disease. Mol. Neurodegener..

[23] O’Brien R.J., Wong P.C. (2011). Amyloid precursor protein processing and Alzheimer’s disease. Annu. Rev. Neurosci..

[24] Hardy J., Selkoe D.J. (2002). The amyloid hypothesis of Alzheimer’s disease: Progress and problems on the road to therapeutics. Science.

[25] D’Errico P., Meyer-Luehmann M. (2020). Mechanisms of Pathogenic Tau and Aβ Protein Spreading in Alzheimer’s Disease. Front. Aging Neurosci..

[26] Bloom G.S. (2014). Amyloid-β and tau: The trigger and bullet in Alzheimer disease pathogenesis. JAMA Neurol..

[27] Zheng W.H., Bastianetto S., Mennicken F., Ma W., Kar S. (2002). Amyloid beta peptide induces tau phosphorylation and loss of cholinergic neurons in rat primary septal cultures. Neuroscience.

[28] Méphon-Gaspard A., Boca M., Pioche-Durieu C., Desforges B., Burgo A., Hamon L., Piétrement O., Pastré D. (2016). Role of tau in the spatial organization of axonal microtubules: Keeping parallel microtubules evenly distributed despite macromolecular crowding. Cell Mol. Life Sci..

[29] John A., Reddy P.H. (2021). Synaptic basis of Alzheimer’s disease: Focus on synaptic amyloid beta, P-tau and mitochondria. Ageing Res. Rev..

[30] Gong C.X., Iqbal K. (2008). Hyperphosphorylation of microtubule-associated protein tau: A promising therapeutic target for Alzheimer disease. Curr. Med. Chem..

[31] Martin L., Latypova X., Wilson C.M., Magnaudeix A., Perrin M.L., Yardin C., Terro F. (2013). Tau protein kinases: Involvement in Alzheimer’s disease. Ageing Res. Rev..

[32] Arnsten A.F.T., Datta D., Del Tredici K., Braak H. (2021). Hypothesis: Tau pathology is an initiating factor in sporadic Alzheimer’s disease. Alzheimers Dement..

[33] Mohandas E., Rajmohan V., Raghunath B. (2009). Neurobiology of Alzheimer’s disease. Indian J. Psychiatry.

[34] Butterfield D.A., Reed T., Newman S.F., Sultana R. (2007). Roles of amyloid beta-peptide-associated oxidative stress and brain protein modifications in the pathogenesis of Alzheimer’s disease and mild cognitive impairment. Free. Radic. Biol. Med..

[35] Butterfield D.A., Lauderback C.M. (2002). Lipid peroxidation and protein oxidation in Alzheimer’s disease brain: Potential causes and consequences involving amyloid beta-peptide-associated free radical oxidative stress. Free. Radic. Biol. Med..

[36] Cheignon C., Tomas M., Bonnefont-Rousselot D., Faller P., Hureau C., Collin F. (2018). Oxidative stress and the amyloid beta peptide in Alzheimer’s disease. Redox. Biol..

[37] Trevisan K., Cristina-Pereira R., Silva-Amaral D., Aversi-Ferreira T.A. (2019). Theories of Aging and the Prevalence of Alzheimer’s Disease. Biomed. Res. Int..

[38] Armstrong R.A. (2019). Risk factors for Alzheimer’s disease. Folia Neuropathol..

[39] Farkas E., Luiten P.G. (2001). Cerebral microvascular pathology in aging and Alzheimer’s disease. Prog. Neurobiol..

[40] Daulatzai M.A. (2017). Cerebral hypoperfusion and glucose hypometabolism: Key pathophysiological modulators promote neurodegeneration, cognitive impairment, and Alzheimer’s disease. J. Neurosci. Res..

[41] Mann K.M., Thorngate F.E., Katoh-Fukui Y., Hamanaka H., Williams D.L., Fujita S., Lamb B.T. (2004). Independent effects of APOE on cholesterol metabolism and brain Abeta levels in an Alzheimer disease mouse model. Hum. Mol. Genet..

[42] Fong L.K., Yang M.M., Dos Santos Chaves R., Reyna S.M., Langness V.F., Woodruff G., Roberts E.A., Young J.E., Goldstein L.S.B. (2018). Full-length amyloid precursor protein regulates lipoprotein metabolism and amyloid-β clearance in human astrocytes. J. Biol. Chem..

[43] Puglielli L., Tanzi R.E., Kovacs D.M. (2003). Alzheimer’s disease: The cholesterol connection. Nat. Neurosci..

[44] Langness V.F., van der Kant R., Das U., Wang L., Chaves R.D.S., Goldstein L.S.B. (2021). Cholesterol-lowering drugs reduce APP processing to Aβ by inducing APP dimerization. Mol. Biol. Cell.

[45] Feringa F.M., van der Kant R. (2021). Cholesterol and Alzheimer’s Disease; From Risk Genes to Pathological Effects. Front. Aging Neurosci..

[46] Tuppo E.E., Arias H.R. (2005). The role of inflammation in Alzheimer’s disease. Int. J. Biochem. Cell Biol..

[47] Moore A.H., O’Banion M.K. (2002). Neuroinflammation and anti-inflammatory therapy for Alzheimer’s disease. Adv. Drug Deliv. Rev..

[48] Michaud M., Balardy L., Moulis G., Gaudin C., Peyrot C., Vellas B., Cesari M., Nourhashemi F. (2013). Proinflammatory cytokines, aging, and age-related diseases. J. Am. Med. Dir. Assoc..

[49] Pluvinage J.V., Haney M.S., Smith B.A.H., Sun J., Iram T., Bonanno L., Li L., Lee D.P., Morgens D.W., Yang A.C. (2019). CD22 blockade restores homeostatic microglial phagocytosis in ageing brains. Nature.

[50] Wang L., Yin Y.L., Liu X.Z., Shen P., Zheng Y.G., Lan X.R., Lu C.B., Wang J.Z. (2020). Current understanding of metal ions in the pathogenesis of Alzheimer’s disease. Transl. Neurodegener..

[51] Das N., Raymick J., Sarkar S. (2021). Role of metals in Alzheimer’s disease. Metab. Brain Dis..

[52] Turab Naqvi A.A., Hasan G.M., Hassan M.I. (2020). Targeting Tau Hyperphosphorylation via Kinase Inhibition: Strategy to Address Alzheimer’s Disease. Curr. Top. Med. Chem..

[53] House E., Collingwood J., Khan A., Korchazkina O., Berthon G., Exley C. (2004). Aluminium, iron, zinc and copper influence the *In vitro* formation of amyloid fibrils of Aβ 42 in a manner which may have consequences for metal chelation therapy in Alzheimer’s disease. J. Alzheimer’s Dis..

[54] Matheou C.J., Younan N.D., Viles J.H. (2015). Cu^2^⁺ accentuates distinct misfolding of Aβ₁₋₄₀ and Aβ₁₋₄₂ peptides, and potentiates membrane disruption. Biochem. J..

[55] Van Duijn S., Bulk M., van Duinen S.G., Nabuurs R.J.A., van Buchem M.A., van der Weerd L., Natté R. (2017). Cortical Iron Reflects Severity of Alzheimer’s Disease. J. Alzheimers Dis..

[56] Breuer W., Ghoti H., Shattat A., Goldfarb A., Koren A., Levin C., Rachmilewitz E., Cabantchik Z.I. (2012). Non-transferrin bound iron in Thalassemia: Differential detection of redox active forms in children and older patients. Am. J. Hematol..

[57] Bush A.I. (2003). The metallobiology of Alzheimer’s disease. Trends Neurosci..

[58] Mezzaroba L., Alfieri D.F., Colado Simão A.N., Vissoci Reiche E.M. (2019). The role of zinc, copper, manganese and iron in neurodegenerative diseases. Neurotoxicology.

[59] Lakey-Beitia J., Burillo A.M., La Penna G., Hegde M.L., Rao K.S. (2021). Polyphenols as Potential Metal Chelation Compounds Against Alzheimer’s Disease. J. Alzheimers Dis..

[60] Liu E.Y.L., Mak S., Kong X., Xia Y., Kwan K.K.L., Xu M.L., Tsim K.W.K. (2021). Tacrine Induces Endoplasmic Reticulum-Stressed Apoptosis via Disrupting the Proper Assembly of Oligomeric Acetylcholinesterase in Cultured Neuronal Cells. Mol. Pharm..

[61] Palmer A.M. (2011). The role of the blood brain barrier in neurodegenerative disorders and their treatment. J. Alzheimers Dis..

[62] Duwa R., Jeong J.-H., Yook S. (2021). Development of immunotherapy and nanoparticles-based strategies for the treatment of Parkinson’s disease. J. Pharm. Investig..

[63] Gonzalez-Carter D., Liu X., Tockary T.A., Dirisala A., Toh K., Anraku Y., Kataoka K. (2020). Targeting nanoparticles to the brain by exploiting the blood-brain barrier impermeability to selectively label the brain endothelium. Proc. Natl. Acad. Sci. USA.

[64] Lee C.S., Leong K.W. (2020). Advances in microphysiological blood-brain barrier (BBB) models towards drug delivery. Curr. Opin. Biotechnol..

[65] Nguyen T.T., Nguyen T.T.D., Nguyen T.K.O., Vo T.K., Vo V.G. (2021). Advances in developing therapeutic strategies for Alzheimer’s disease. Biomed. Pharmacother..

[66] Nguyen T.T., Vo T.K., Vo G.V. (2021). Therapeutic Strategies and Nano-Drug Delivery Applications in Management of Aging Alzheimer’s Disease. Adv. Exp. Med. Biol..

[67] Béduneau A., Saulnier P., Benoit J.P. (2007). Active targeting of brain tumors using nanocarriers. Biomaterials.

[68] Han L., Jiang C. (2021). Evolution of blood-brain barrier in brain diseases and related systemic nanoscale brain-targeting drug delivery strategies. Acta Pharm. Sin. B.

[69] Zhou Y., Peng Z., Seven E.S., Leblanc R.M. (2018). Crossing the blood-brain barrier with nanoparticles. J. Control. Release.

[70] Kumagai A., Eisenberg J.B., Pardridge W. (1987). Absorptive-mediated endocytosis of cationized albumin and a beta-endorphin-cationized albumin chimeric peptide by isolated brain capillaries. Model system of blood-brain barrier transport. J. Biol. Chem..

[71] Hervé F., Ghinea N., Scherrmann J.M. (2008). CNS delivery via adsorptive transcytosis. Aaps J..

[72] Song J., Lu C., Leszek J., Zhang J. (2021). Design and Development of Nanomaterial-Based Drug Carriers to Overcome the Blood-Brain Barrier by Using Different Transport Mechanisms. Int. J. Mol. Sci..

[73] Zheng P.P., Romme E., van der Spek P.J., Dirven C.M., Willemsen R., Kros J.M. (2010). Glut1/SLC2A1 is crucial for the development of the blood-brain barrier *in vivo*. Ann. Neurol..

[74] Huttunen J., Peltokangas S., Gynther M., Natunen T., Hiltunen M., Auriola S., Ruponen M., Vellonen K.S., Huttunen K.M. (2019). L-Type Amino Acid Transporter 1 (LAT1/Lat1)-Utilizing Prodrugs Can Improve the Delivery of Drugs into Neurons, Astrocytes and Microglia. Sci. Rep..

[75] Akanuma S.I. (2020). [Membrane Transporters and Their Regulatory Mechanisms at the Brain and Retinal Barriers to Establish Therapies for Refractory Central Nervous System Diseases]. Yakugaku Zasshi.

[76] Jones A.R., Shusta E.V. (2007). Blood-brain barrier transport of therapeutics via receptor-mediation. Pharm. Res..

[77] Brasnjevic I., Steinbusch H.W., Schmitz C., Martinez-Martinez P. (2009). Delivery of peptide and protein drugs over the blood-brain barrier. Prog. Neurobiol..

[78] Scheld W.M. (1989). Drug delivery to the central nervous system: General principles and relevance to therapy for infections of the central nervous system. Rev. Infect. Dis..

[79] Zhu J., Zhang Y., Chen X., Zhang Y., Zhang K., Zheng H., Wei Y., Zheng H., Zhu J., Wu F. (2021). Angiopep-2 modified lipid-coated mesoporous silica nanoparticles for glioma targeting therapy overcoming BBB. Biochem. Biophys. Res. Commun..

[80] Yurek D.M., Hasselrot U., Cass W.A., Sesenoglu-Laird O., Padegimas L., Cooper M.J. (2015). Age and lesion-induced increases of GDNF transgene expression in brain following intracerebral injections of DNA nanoparticles. Neuroscience.

[81] Noor N.A., Hosny E.N., Khadrawy Y.A., Mourad I.M., Othman A.I., Aboul Ezz H.S., Mohammed H.S. (2022). Effect of curcumin nanoparticles on streptozotocin-induced male Wistar rat model of Alzheimer’s disease. Metab. Brain Dis..

[82] Heydari S., Hedayati Ch M., Saadat F., Abedinzade M., Nikokar I., Aboutaleb E., Khafri A., Mokarram A.R. (2020). Diphtheria toxoid nanoparticles improve learning and memory impairment in animal model of Alzheimer’s disease. Pharm. Rep..

[83] Mahdi O., Baharuldin M.T.H., Nor N.H.M., Chiroma S.M., Jagadeesan S., Moklas M.A.M. (2019). Chemicals used for the induction of Alzheimer’s disease-like cognitive dysfunctions in rodents. Biomed. Res. Ther..

[84] Householder K.T., Dharmaraj S., Sandberg D.I., Wechsler-Reya R.J., Sirianni R.W. (2019). Fate of nanoparticles in the central nervous system after intrathecal injection in healthy mice. Sci. Rep..

[85] Dai H., Navath R.S., Balakrishnan B., Guru B.R., Mishra M.K., Romero R., Kannan R.M., Kannan S. (2010). Intrinsic targeting of inflammatory cells in the brain by polyamidoamine dendrimers upon subarachnoid administration. Nanomedicine.

[86] Formicola B., Cox A., Dal Magro R., Masserini M., Re F. (2019). Nanomedicine for the Treatment of Alzheimer’s Disease. J. Biomed. Nanotechnol..

[87] Islam S.U., Shehzad A., Ahmed M.B., Lee Y.S. (2020). Intranasal Delivery of Nanoformulations: A Potential Way of Treatment for Neurological Disorders. Molecules.

[88] Prabakaran A., Agrawal M., Dethe M.R., Ahmed H., Yadav A., Gupta U., Alexander A. (2022). Nose-to-brain drug delivery for the treatment of Alzheimer’s disease: Current advancements and challenges. Expert. Opin. Drug Deliv..

[89] Fonseca L.C., Lopes J.A., Vieira J., Viegas C., Oliveira C.S., Hartmann R.P., Fonte P. (2021). Intranasal drug delivery for treatment of Alzheimer’s disease. Drug Deliv. Transl. Res..

[90] Bhatt P.C., Srivastava P., Pandey P., Khan W., Panda B.P. (2016). Nose to brain delivery of astaxanthin-loaded solid lipid nanoparticles: Fabrication, radio labeling, optimization and biological studies. RSC Adv..

[91] Lungare S., Hallam K., Badhan R.K.S. (2016). Phytochemical-loaded mesoporous silica nanoparticles for nose-to-brain olfactory drug delivery. Int. J. Pharm..

[92] Muntimadugu E., Dhommati R., Jain A., Challa V.G., Shaheen M., Khan W. (2016). Intranasal delivery of nanoparticle encapsulated tarenflurbil: A potential brain targeting strategy for Alzheimer’s disease. Eur. J. Pharm. Sci..

[93] Wu S.K., Tsai C.L., Huang Y., Hynynen K. (2020). Focused Ultrasound and Microbubbles-Mediated Drug Delivery to Brain Tumor. Pharmaceutics.

[94] Burgess A., Shah K., Hough O., Hynynen K. (2015). Focused ultrasound-mediated drug delivery through the blood-brain barrier. Expert. Rev. Neurother..

[95] Liu Y., Gong Y., Xie W., Huang A., Yuan X., Zhou H., Zhu X., Chen X., Liu J., Liu J. (2020). Microbubbles in combination with focused ultrasound for the delivery of quercetin-modified sulfur nanoparticles through the blood brain barrier into the brain parenchyma and relief of endoplasmic reticulum stress to treat Alzheimer’s disease. Nanoscale.

[96] Kofoed R.H., Heinen S., Silburt J., Dubey S., Dibia C.L., Maes M., Simpson E.M., Hynynen K., Aubert I. (2021). Transgene distribution and immune response after ultrasound delivery of rAAV9 and PHP.B to the brain in a mouse model of amyloidosis. Mol. Methods Clin. Dev..

[97] Cheng C.S., Liu T.P., Chien F.C., Mou C.Y., Wu S.H., Chen Y.P. (2019). Codelivery of Plasmid and Curcumin with Mesoporous Silica Nanoparticles for Promoting Neurite Outgrowth. ACS Appl. Mater. Interfaces.

[98] Raymond J.J., Robertson D.M., Dinsdale H.B. (1986). Pharmacological modification of bradykinin induced breakdown of the blood-brain barrier. Can. J. Neurol. Sci..

[99] Praça C., Rai A., Santos T., Cristovão A.C., Pinho S.L., Cecchelli R., Dehouck M.P., Bernardino L., Ferreira L.S. (2018). A nanoformulation for the preferential accumulation in adult neurogenic niches. J. Control. Release.

[100] Picone P., Ditta L.A., Sabatino M.A., Militello V., San Biagio P.L., Di Giacinto M.L., Cristaldi L., Nuzzo D., Dispenza C., Giacomazza D. (2016). Ionizing radiation-engineered nanogels as insulin nanocarriers for the development of a new strategy for the treatment of Alzheimer’s disease. Biomaterials.

[101] Zhou H., Gong Y., Liu Y., Huang A., Zhu X., Liu J., Yuan G., Zhang L., Wei J.A., Liu J. (2020). Intelligently thermoresponsive flower-like hollow nano-ruthenium system for sustained release of nerve growth factor to inhibit hyperphosphorylation of tau and neuronal damage for the treatment of Alzheimer’s disease. Biomaterials.

[102] Liu W., Wang W., Dong X., Sun Y. (2020). Near-Infrared Light-Powered Janus Nanomotor Significantly Facilitates Inhibition of Amyloid-β Fibrillogenesis. ACS Appl. Mater. Interfaces.

[103] Yamazaki Y., Kanekiyo T. (2017). Blood-Brain Barrier Dysfunction and the Pathogenesis of Alzheimer’s Disease. Int. J. Mol. Sci..

[104] Honary S., Zahir F. (2013). Effect of zeta potential on the properties of nano-drug delivery systems-a review (Part 1). Trop. J. Pharm. Res..

[105] Zhang S., Gao H., Bao G. (2015). Physical Principles of Nanoparticle Cellular Endocytosis. ACS Nano.

[106] Sukhanova A., Bozrova S., Sokolov P., Berestovoy M., Karaulov A., Nabiev I. (2018). Dependence of nanoparticle toxicity on their physical and chemical properties. Nanoscale Res. Lett..

[107] Li W., Zhou Y., Zhao N., Hao B., Wang X., Kong P. (2012). Pharmacokinetic behavior and efficiency of acetylcholinesterase inhibition in rat brain after intranasal administration of galanthamine hydrobromide loaded flexible liposomes. Environ. Toxicol. Pharm..

[108] Yang Z.Z., Zhang Y.Q., Wang Z.Z., Wu K., Lou J.N., Qi X.R. (2013). Enhanced brain distribution and pharmacodynamics of rivastigmine by liposomes following intranasal administration. Int. J. Pharm..

[109] Al Asmari A.K., Ullah Z., Tariq M., Fatani A. (2016). Preparation, characterization, and *in vivo* evaluation of intranasally administered liposomal formulation of donepezil. Drug Des. Devel..

[110] Ordóñez-Gutiérrez L., Wandosell F. (2020). Nanoliposomes as a Therapeutic Tool for Alzheimer’s Disease. Front. Synaptic. Neurosci..

[111] Arora S., Layek B., Singh J. (2021). Design and Validation of Liposomal ApoE2 Gene Delivery System to Evade Blood-Brain Barrier for Effective Treatment of Alzheimer’s Disease. Mol. Pharm..

[112] Saffari P.M., Alijanpour S., Takzaree N., Sahebgharani M., Etemad-Moghadam S., Noorbakhsh F., Partoazar A. (2020). Metformin loaded phosphatidylserine nanoliposomes improve memory deficit and reduce neuroinflammation in streptozotocin-induced Alzheimer’s disease model. Life Sci..

[113] Rotman M., Welling M.M., Bunschoten A., de Backer M.E., Rip J., Nabuurs R.J., Gaillard P.J., van Buchem M.A., van der Maarel S.M., van der Weerd L. (2015). Enhanced glutathione PEGylated liposomal brain delivery of an anti-amyloid single domain antibody fragment in a mouse model for Alzheimer’s disease. J. Control. Release.

[114] Kong L., Li X.T., Ni Y.N., Xiao H.H., Yao Y.J., Wang Y.Y., Ju R.J., Li H.Y., Liu J.J., Fu M. (2020). Transferrin-Modified Osthole PEGylated Liposomes Travel the Blood-Brain Barrier and Mitigate Alzheimer’s Disease-Related Pathology in APP/PS-1 Mice. Int. J. Nanomed..

[115] Pardridge W.M. (2020). Brain Delivery of Nanomedicines: Trojan Horse Liposomes for Plasmid DNA Gene Therapy of the Brain. Front. Med. Technol..

[116] Pardridge W.M. (2010). Preparation of Trojan horse liposomes (THLs) for gene transfer across the blood-brain barrier. Cold Spring Harb. Protoc..

[117] Kocsis I., Sanna E., Hunter C.A. (2021). Liposome Enhanced Detection of Amyloid Protein Aggregates. Org. Lett..

[118] Yang P., Sheng D., Guo Q., Wang P., Xu S., Qian K., Li Y., Cheng Y., Wang L., Lu W. (2020). Neuronal mitochondria-targeted micelles relieving oxidative stress for delayed progression of Alzheimer’s disease. Biomaterials.

[119] Candela P., Gosselet F., Saint-Pol J., Sevin E., Boucau M.C., Boulanger E., Cecchelli R., Fenart L. (2010). Apical-to-basolateral transport of amyloid-β peptides through blood-brain barrier cells is mediated by the receptor for advanced glycation end-products and is restricted by P-glycoprotein. J. Alzheimers Dis..

[120] Lu Y., Guo Z., Zhang Y., Li C., Zhang Y., Guo Q., Chen Q., Chen X., He X., Liu L. (2019). Microenvironment Remodeling Micelles for Alzheimer’s Disease Therapy by Early Modulation of Activated Microglia. Adv. Sci..

[121] Agwa M.M., Abdelmonsif D.A., Khattab S.N., Sabra S. (2020). Self- assembled lactoferrin-conjugated linoleic acid micelles as an orally active targeted nanoplatform for Alzheimer’s disease. Int. J. Biol. Macromol..

[122] Geng H., Yuan H., Qiu L., Gao D., Cheng Y., Xing C. (2020). Inhibition and disaggregation of amyloid β protein fibrils through conjugated polymer-core thermoresponsive micelles. J. Mater. Chem. B.

[123] Misra S., Chopra K., Sinha V.R., Medhi B. (2016). Galantamine-loaded solid-lipid nanoparticles for enhanced brain delivery: Preparation, characterization, *In vitro* and *in vivo* evaluations. Drug Deliv..

[124] Shah B., Khunt D., Bhatt H., Misra M., Padh H. (2015). Application of quality by design approach for intranasal delivery of rivastigmine loaded solid lipid nanoparticles: Effect on formulation and characterization parameters. Eur. J. Pharm. Sci..

[125] Chauhan M.K., Sharma P.K. (2019). Optimization and characterization of rivastigmine nanolipid carrier loaded transdermal patches for the treatment of dementia. Chem. Phys. Lipids.

[126] Mendes I.T., Ruela A.L.M., Carvalho F.C., Freitas J.T.J., Bonfilio R., Pereira G.R. (2019). Development and characterization of nanostructured lipid carrier-based gels for the transdermal delivery of donepezil. Colloids Surf. B Biointerfaces.

[127] Dara T., Vatanara A., Sharifzadeh M., Khani S., Vakilinezhad M.A., Vakhshiteh F., Nabi Meybodi M., Sadegh Malvajerd S., Hassani S., Mosaddegh M.H. (2019). Improvement of memory deficits in the rat model of Alzheimer’s disease by erythropoietin-loaded solid lipid nanoparticles. Neurobiol. Learn. Mem..

[128] Vakilinezhad M.A., Amini A., Akbari Javar H., Baha’addini Beigi Zarandi B.F., Montaseri H., Dinarvand R. (2018). Nicotinamide loaded functionalized solid lipid nanoparticles improves cognition in Alzheimer’s disease animal model by reducing Tau hyperphosphorylation. Daru.

[129] Yadav A., Sunkaria A., Singhal N., Sandhir R. (2018). Resveratrol loaded solid lipid nanoparticles attenuate mitochondrial oxidative stress in vascular dementia by activating Nrf2/HO-1 pathway. Neurochem. Int..

[130] Loureiro J.A., Andrade S., Duarte A., Neves A.R., Queiroz J.F., Nunes C., Sevin E., Fenart L., Gosselet F., Coelho M.A.N. (2017). Resveratrol and Grape Extract-loaded Solid Lipid Nanoparticles for the Treatment of Alzheimer’s Disease. Molecules.

[131] Pinheiro R.G.R., Granja A., Loureiro J.A., Pereira M.C., Pinheiro M., Neves A.R., Reis S. (2020). Quercetin lipid nanoparticles functionalized with transferrin for Alzheimer’s disease. Eur. J. Pharm. Sci..

[132] Dhawan S., Kapil R., Singh B. (2011). Formulation development and systematic optimization of solid lipid nanoparticles of quercetin for improved brain delivery. J. Pharm. Pharm..

[133] Meng F., Asghar S., Gao S., Su Z., Song J., Huo M., Meng W., Ping Q., Xiao Y. (2015). A novel LDL-mimic nanocarrier for the targeted delivery of curcumin into the brain to treat Alzheimer’s disease. Colloids Surf. B: Biointerfaces.

[134] Zielińska A., Carreiró F., Oliveira A.M., Neves A., Pires B., Venkatesh D.N., Durazzo A., Lucarini M., Eder P., Silva A.M. (2020). Polymeric Nanoparticles: Production, Characterization, Toxicology and Ecotoxicology. Molecules.

[135] Allahyari M., Mohit E. (2016). Peptide/protein vaccine delivery system based on PLGA particles. Hum. Vaccin Immunother..

[136] Baysal I., Ucar G., Gultekinoglu M., Ulubayram K., Yabanoglu-Ciftci S. (2017). Donepezil loaded PLGA-b-PEG nanoparticles: Their ability to induce destabilization of amyloid fibrils and to cross blood brain barrier *In vitro*. J. Neural. Transm..

[137] Nanaki S.G., Spyrou K., Bekiari C., Veneti P., Baroud T.N., Karouta N., Grivas I., Papadopoulos G.C., Gournis D., Bikiaris D.N. (2020). Hierarchical Porous Carbon-PLLA and PLGA Hybrid Nanoparticles for Intranasal Delivery of Galantamine for Alzheimer’s Disease Therapy. Pharmaceutics.

[138] Pagar K., Vavia P. (2013). Rivastigmine-loaded L-lactide-depsipeptide polymeric nanoparticles: Decisive formulation variable optimization. Sci. Pharm..

[139] Yin H., Si J., Xu H., Dong J., Zheng D., Lu X., Li X. (2014). Resveratrol-loaded nanoparticles reduce oxidative stress induced by radiation or amyloid-beta in transgenic Caenorhabditis elegans. J. Biomed. Nanotechnol..

[140] Fan S., Zheng Y., Liu X., Fang W., Chen X., Liao W., Jing X., Lei M., Tao E., Ma Q. (2018). Curcumin-loaded PLGA-PEG nanoparticles conjugated with B6 peptide for potential use in Alzheimer’s disease. Drug Deliv..

[141] Liu Z., Gao X., Kang T., Jiang M., Miao D., Gu G., Hu Q., Song Q., Yao L., Tu Y. (2013). B6 peptide-modified PEG-PLA nanoparticles for enhanced brain delivery of neuroprotective peptide. Bioconjug. Chem..

[142] Cano A., Ettcheto M., Chang J.H., Barroso E., Espina M., Kühne B.A., Barenys M., Auladell C., Folch J., Souto E.B. (2019). Dual-drug loaded nanoparticles of Epigallocatechin-3-gallate (EGCG)/Ascorbic acid enhance therapeutic efficacy of EGCG in a APPswe/PS1dE9 Alzheimer’s disease mice model. J. Control. Release.

[143] Silva-Abreu M., Calpena A.C., Andrés-Benito P., Aso E., Romero I.A., Roig-Carles D., Gromnicova R., Espina M., Ferrer I., García M.L. (2018). PPARγ agonist-loaded PLGA-PEG nanocarriers as a potential treatment for Alzheimer’s disease: *In vitro* and *in vivo* studies. Int. J. Nanomed..

[144] Sun D., Li N., Zhang W., Zhao Z., Mou Z., Huang D., Liu J., Wang W. (2016). Design of PLGA-functionalized quercetin nanoparticles for potential use in Alzheimer’s disease. Colloids Surf. B Biointerfaces.

[145] Manek E., Darvas F., Petroianu G.A. (2020). Use of Biodegradable, Chitosan-Based Nanoparticles in the Treatment of Alzheimer’s Disease. Molecules.

[146] Singh S.K., Mishra D.N. (2019). Nose to Brain Delivery of Galantamine Loaded Nanoparticles: In-vivo Pharmacodynamic and Biochemical Study in Mice. Curr. Drug Deliv..

[147] Mahl C.R.A., Taketa T.B., Rocha-Neto J.B.M., Almeida W.P., Beppu M.M. (2020). Copper Ion Uptake by Chitosan in the Presence of Amyloid-β and Histidine. Appl. Biochem. Biotechnol..

[148] Gothwal A., Kumar H., Nakhate K.T., Ajazuddin, Dutta A., Borah A., Gupta U. (2019). Lactoferrin Coupled Lower Generation PAMAM Dendrimers for Brain Targeted Delivery of Memantine in Aluminum-Chloride-Induced Alzheimer’s Disease in Mice. Bioconjug Chem..

[149] DeRidder L., Sharma A., Liaw K., Sharma R., John J., Kannan S., Kannan R.M. (2021). Dendrimer-tesaglitazar conjugate induces a phenotype shift of microglia and enhances β-amyloid phagocytosis. Nanoscale.

[150] Klajnert B., Wasiak T., Ionov M., Fernandez-Villamarin M., Sousa-Herves A., Correa J., Riguera R., Fernandez-Megia E. (2012). Dendrimers reduce toxicity of Aβ 1-28 peptide during aggregation and accelerate fibril formation. Nanomedicine.

[151] Gupta A., Chung E.J., Leon L., Rinaldi C. (2020). Chapter 21—Nanoemulsions. Nanoparticles for Biomedical Applications.

[152] Altinoglu G., Adali T. (2020). Alzheimer’s Disease Targeted Nano-Based Drug Delivery Systems. Curr. Drug Targets.

[153] Nirale P., Paul A., Yadav K.S. (2020). Nanoemulsions for targeting the neurodegenerative diseases: Alzheimer’s, Parkinson’s and Prion’s. Life Sci..

[154] Kaur A., Nigam K., Srivastava S., Tyagi A., Dang S. (2020). Memantine nanoemulsion: A new approach to treat Alzheimer’s disease. J. Microencapsul..

[155] Kaur A., Nigam K., Bhatnagar I., Sukhpal H., Awasthy S., Shankar S., Tyagi A., Dang S. (2020). Treatment of Alzheimer’s diseases using donepezil nanoemulsion: An intranasal approach. Drug Deliv. Transl. Res..

[156] Jiang Y., Liu C., Zhai W., Zhuang N., Han T., Ding Z. (2019). The Optimization Design Of Lactoferrin Loaded HupA Nanoemulsion For Targeted Drug Transport Via Intranasal Route. Int. J. Nanomed..

[157] Li X., Tsibouklis J., Weng T., Zhang B., Yin G., Feng G., Cui Y., Savina I.N., Mikhalovska L.I., Sandeman S.R. (2017). Nano carriers for drug transport across the blood-brain barrier. J. Drug Target.

[158] Tak K., Sharma R., Dave V., Jain S., Sharma S. (2020). Clitoria ternatea Mediated Synthesis of Graphene Quantum Dots for the Treatment of Alzheimer’s Disease. ACS Chem. Neurosci..

[159] Wang S., Li C., Xia Y., Chen S., Robert J., Banquy X., Huang R., Qi W., He Z., Su R. (2020). Nontoxic Black Phosphorus Quantum Dots Inhibit Insulin Amyloid Fibrillation at an Ultralow Concentration. iScience.

[160] Zhou X., Hu S., Wang S., Pang Y., Lin Y., Li M. (2021). Large Amino Acid Mimicking Selenium-Doped Carbon Quantum Dots for Multi-Target Therapy of Alzheimer’s Disease. Front. Pharm..

[161] Mars A., Hamami M., Bechnak L., Patra D., Raouafi N. (2018). Curcumin-graphene quantum dots for dual mode sensing platform: Electrochemical and fluorescence detection of APOe4, responsible of Alzheimer’s disease. Anal. Chim. Acta.

[162] Hou K., Zhao J., Wang H., Li B., Li K., Shi X., Wan K., Ai J., Lv J., Wang D. (2020). Chiral gold nanoparticles enantioselectively rescue memory deficits in a mouse model of Alzheimer’s disease. Nat. Commun..

[163] Dos Santos Tramontin N., da Silva S., Arruda R., Ugioni K.S., Canteiro P.B., de Bem Silveira G., Mendes C., Silveira P.C.L., Muller A.P. (2020). Gold Nanoparticles Treatment Reverses Brain Damage in Alzheimer’s Disease Model. Mol. Neurobiol..

[164] Sanati M., Khodagholi F., Aminyavari S., Ghasemi F., Gholami M., Kebriaeezadeh A., Sabzevari O., Hajipour M.J., Imani M., Mahmoudi M. (2019). Impact of Gold Nanoparticles on Amyloid β-Induced Alzheimer’s Disease in a Rat Animal Model: Involvement of STIM Proteins. ACS Chem. Neurosci..

[165] Razzino C.A., Serafín V., Gamella M., Pedrero M., Montero-Calle A., Barderas R., Calero M., Lobo A.O., Yáñez-Sedeño P., Campuzano S. (2020). An electrochemical immunosensor using gold nanoparticles-PAMAM-nanostructured screen-printed carbon electrodes for tau protein determination in plasma and brain tissues from Alzheimer patients. Biosens. Bioelectron..

[166] Yin L., Wang Y., Tan R., Li H., Tu Y. (2021). Determination of β-amyloid oligomer using electrochemiluminescent aptasensor with signal enhancement by AuNP/MOF nanocomposite. Mikrochim. Acta.

[167] Enteshari Najafabadi R., Kazemipour N., Esmaeili A., Beheshti S., Nazifi S. (2018). Using superparamagnetic iron oxide nanoparticles to enhance bioavailability of quercetin in the intact rat brain. BMC Pharm. Toxicol..

[168] Li Y., Lim E., Fields T., Wu H., Xu Y., Wang Y.A., Mao H. (2019). Improving Sensitivity and Specificity of Amyloid-β Peptides and Tau Protein Detection with Antibiofouling Magnetic Nanoparticles for Liquid Biopsy of Alzheimer’s Disease. ACS Biomater. Sci. Eng..

[169] Antonoglou O., Giannousi K., Mourdikoudis S., Dendrinou-Samara C. (2020). Magnetic nanoemulsions as candidates for Alzheimer’s disease dual imaging theranostics. Nanotechnology.

[170] Yin T., Yang L., Liu Y., Zhou X., Sun J., Liu J. (2015). Sialic acid (SA)-modified selenium nanoparticles coated with a high blood-brain barrier permeability peptide-B6 peptide for potential use in Alzheimer’s disease. Acta Biomater..

[171] Lopez-Barbosa N., Garcia J.G., Cifuentes J., Castro L.M., Vargas F., Ostos C., Cardona-Gomez G.P., Hernandez A.M., Cruz J.C. (2020). Multifunctional magnetite nanoparticles to enable delivery of siRNA for the potential treatment of Alzheimer’s. Drug Deliv..

[172] Morgan D. (2011). Immunotherapy for Alzheimer’s disease. J. Intern. Med..

[173] Schenk D. (2002). Amyloid-β immunotherapy for Alzheimer’s disease: The end of the beginning. Nat. Rev. Neurosci..

[174] Sarlus H., Heneka M.T. (2017). Microglia in Alzheimer’s disease. J. Clin. Investig..

[175] Hansen D.V., Hanson J.E., Sheng M. (2018). Microglia in Alzheimer’s disease. J. Cell Biol..

[176] Fassbender K., Walter S., Kühl S., Landmann R., Ishii K., Bertsch T., Stalder A., Muehlhauser F., Liu Y., Ulmer A. (2004). The LPS receptor (CD14) links innate immunity with Alzheimer’s disease. FASEB J..

[177] Hardy J., Duff K., Hardy K.G., Perez-Tur J., Hutton M. (1998). Genetic dissection of Alzheimer’s disease and related dementias: Amyloid and its relationship to tau. Nat. Neurosci..

[178] Ittner A., Bertz J., Suh L.S., Stevens C.H., Götz J., Ittner L.M. (2015). Tau-targeting passive immunization modulates aspects of pathology in tau transgenic mice. J. Neurochem..

[179] Baruch K., Deczkowska A., Rosenzweig N., Tsitsou-Kampeli A., Sharif A.M., Matcovitch-Natan O., Kertser A., David E., Amit I., Schwartz M. (2016). PD-1 immune checkpoint blockade reduces pathology and improves memory in mouse models of Alzheimer’s disease. Nat. Med..

[180] Schwartz M. (2017). Can immunotherapy treat neurodegeneration?. Science.

[181] Rosenzweig N., Dvir-Szternfeld R., Tsitsou-Kampeli A., Keren-Shaul H., Ben-Yehuda H., Weill-Raynal P., Cahalon L., Kertser A., Baruch K., Amit I. (2019). PD-1/PD-L1 checkpoint blockade harnesses monocyte-derived macrophages to combat cognitive impairment in a tauopathy mouse model. Nat. Commun..

[182] Latta-Mahieu M., Elmer B., Bretteville A., Wang Y., Lopez-Grancha M., Goniot P., Moindrot N., Ferrari P., Blanc V., Schussler N. (2018). Systemic immune-checkpoint blockade with anti-PD1 antibodies does not alter cerebral amyloid-β burden in several amyloid transgenic mouse models. Glia.

[183] Obst J., Mancuso R., Simon E., Gomez-Nicola D. (2018). PD-1 deficiency is not sufficient to induce myeloid mobilization to the brain or alter the inflammatory profile during chronic neurodegeneration. Brain Behav. Immun..

[184] Cai Z., Liu N., Wang C., Qin B., Zhou Y., Xiao M., Chang L., Yan L.J., Zhao B. (2016). Role of RAGE in Alzheimer’s Disease. Cell Mol. Neurobiol..

[185] Fang F., Lue L.F., Yan S., Xu H., Luddy J.S., Chen D., Walker D.G., Stern D.M., Yan S., Schmidt A.M. (2010). RAGE-dependent signaling in microglia contributes to neuroinflammation, Abeta accumulation, and impaired learning/memory in a mouse model of Alzheimer’s disease. FASEB J..

[186] Kong Y., Liu C., Zhou Y., Qi J., Zhang C., Sun B., Wang J., Guan Y. (2020). Progress of RAGE Molecular Imaging in Alzheimer’s Disease. Front. Aging Neurosci..

[187] Loureiro J.A., Gomes B., Fricker G., Cardoso I., Ribeiro C.A., Gaiteiro C., Coelho M.A., Pereira Mdo C., Rocha S. (2015). Dual ligand immunoliposomes for drug delivery to the brain. Colloids Surf. B Biointerfaces.

[188] Hao S., Li X., Han A., Yang Y., Fang G., Liu J., Wang S. (2019). CLVFFA-Functionalized Gold Nanoclusters Inhibit Aβ40 Fibrillation, Fibrils’ Prolongation, and Mature Fibrils’ Disaggregation. ACS Chem. Neurosci..

[189] Skaat H., Corem-Slakmon E., Grinberg I., Last D., Goez D., Mardor Y., Margel S. (2013). Antibody-conjugated, dual-modal, near-infrared fluorescent iron oxide nanoparticles for antiamyloidgenic activity and specific detection of amyloid-β fibrils. Int. J. Nanomed..

[190] Chen L., Lin J., Yi J., Weng Q., Zhou Y., Han Z., Li C., Chen J., Zhang Q. (2019). A tyrosinase-induced fluorescence immunoassay for detection of tau protein using dopamine-functionalized CuInS(2)/ZnS quantum dots. Anal. Bioanal. Chem..

[191] Lo Buono N., Parrotta R., Morone S., Bovino P., Nacci G., Ortolan E., Horenstein A.L., Inzhutova A., Ferrero E., Funaro A. (2011). The CD157-integrin partnership controls transendothelial migration and adhesion of human monocytes. J. Biol. Chem..

[192] Wimmer I., Tietz S., Nishihara H., Deutsch U., Sallusto F., Gosselet F., Lyck R., Muller W.A., Lassmann H., Engelhardt B. (2019). PECAM-1 Stabilizes Blood-Brain Barrier Integrity and Favors Paracellular T-Cell Diapedesis Across the Blood-Brain Barrier During Neuroinflammation. Front. Immunol..

[193] Chang X., Li J., Niu S., Xue Y., Tang M. (2021). Neurotoxicity of metal-containing nanoparticles and implications in glial cells. J. Appl. Toxicol..

[194] Hu Y.L., Gao J.Q. (2010). Potential neurotoxicity of nanoparticles. Int. J. Pharm..

